# Ferroptosis is induced following siramesine and lapatinib treatment of breast cancer cells

**DOI:** 10.1038/cddis.2016.208

**Published:** 2016-07-21

**Authors:** S Ma, E S Henson, Y Chen, S B Gibson

**Affiliations:** 1 Research Institute in Oncology and Hematology, CancerCare Manitoba, University of Manitoba, Winnipeg, MB, Canada R3E OV9; 2 Department of Biochemistry and Medical Genetics, University of Manitoba, Winnipeg, MB, Canada R3E 0J9; 3 Key Laboratory of Radiobiology (Ministry of Health), School of Public Health, Jilin University, 1163 Xinmin Street, Changchun 130021, Jilin, China

## Abstract

Ferroptosis is an iron-dependent, oxidative cell death, and is distinct from apoptosis, necrosis and autophagy. In this study, we demonstrated that lysosome disrupting agent, siramesine and a tyrosine kinase inhibitor, lapatinib synergistically induced cell death and reactive oxygen species (ROS) in MDA MB 231, MCF-7, ZR-75 and SKBr3 breast cancer cells over a 24 h time course. Furthermore, the iron chelator deferoxamine (DFO) significantly reduced cytosolic ROS and cell death following treatment with siramesine and lapatinib. Furthermore, we determined that FeCl3 levels were elevated in cells treated with siramesine and lapatinib indicating an iron-dependent cell death, ferroptosis. To confirm this, we treated cells with a potent inhibitor of ferroptosis, ferrastatin-1 that effectively inhibited cell death following siramesine and lapatinib treatment. The increase levels of iron could be due to changes in iron transport. We found that the expression of transferrin, which is responsible for the transport of iron into cells, is increased following treatment with lapatinib alone or in combination with siramesine. Knocking down of transferrin resulted in decreased cell death and ROS after treatment. In addition, ferroportin-1 (FPN) is an iron transport protein, responsible for removal of iron from cells. We found its expression is decreased after treatment with siramesine alone or in combination with lapatinib. Overexpression FPN resulted in decreased ROS and cell death whereas knockdown of FPN increased cell death after siramesine and lapatinib treatment. This indicates a novel induction of ferroptosis through altered iron regulation by treating breast cancer cells with a lysosome disruptor and a tyrosine kinase inhibitor.

Ferroptotic cell death is a type of cell death that is morphologically, biochemically and genetically distinct from apoptosis, various forms of necrosis, and autophagy.^1, 2^ This process is characterized by iron-dependent accumulation of reactive oxygen species (ROS). Unlike other forms of apoptotic and non-apoptotic death,^3, 4^ this requirement for ROS accumulation appears to be universal. Several genes or proteins responsible for the regulation of iron and ROS metabolism have been implicated in ferroptosis, but the mechanisms to induce and regulate ferroptosis in breast cancer cells remains largely unknown.

Lysosomotropic agents are drugs that destabilize the lysosome membrane directly causing leakage of lysosomal content within the cell.^5^ Siramesine is a sigma-2 receptor ligand that was a lysosomotropic agent and originally developed for treatment of depression.^6^ Although clinical trials failed to show significant efficacy in patients, there are no toxic side effects. In a variety of cancer cells including breast cancer cells, siramesine was shown to induce cell death. It was further shown to induce a rapid rise in the lysosomal pH followed by lysosomal leakage mediated in part by inhibiting sphingomyelinase (ASM). This destabilizing of lysosome membranes led to cathepsin B release and increased ROS causing cell death. Siramesine-induced cell death was independent of the activation of known caspase cascades since siramesine failed to induce detectable caspase activation and the pharmacologic caspase inhibitor z-VAD-fmk could not block the cell death.^7^ Lapatinib is a dual tyrosine kinase inhibitor of ErbB1 and ErbB2 tyrosine kinase receptors. Lapatinib has been approved for treatment of ErbB2-positive breast cancer and for other cancers that overexpress ErbB2. In particular, it was adopted as a therapeutic agent for the treatment of patients with ErbB2-positive refractory advanced or metastatic breast cancer, who had received previous failed treatments such as trastuzumab, anthracyclines and taxanes.^8, 9^ *In vitro* and *in vivo* studies demonstrated that lapatinib was able to inhibit proliferation of ErbB2 and epidermal growth factor receptor-overexpressing cancer cells and induce apoptosis.^8, 9, 10^ Although lapatinib provides a new treatment option for ErbB2-positive cancer, lapatinib monotherapy frequently demonstrated only modest activity in intermediate ErbB2-positive breast cancer cells.^11^ In this study, we investigated the synergic effects of siramesine and lapatinib on cell death in breast cancer cell lines, and the role of iron regulatory proteins and ROS in regulating ferroptosis in breasts cancer cells.

## Results

### Siramesine and lapatinib-induced synergistic cell death

**Figure 1:**
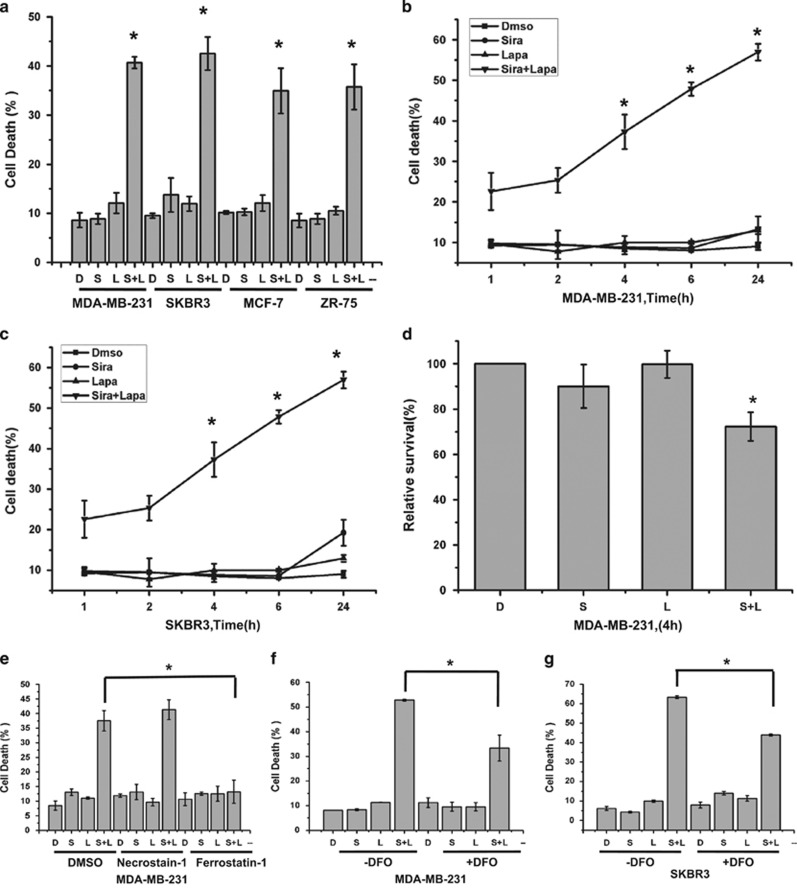
Siramesine and lapatinib-induced synergic cell death. (**a**) MDA-MB 231, SKBR3, MCF-7 and ZR-75-1 breast cancer cells treated for 4 h, respectively, cell death was quantified by trypan blue exclusion assay using flow cytometry as described in the Materials and Methods section. (**b**,**c**) MDA MB-231 and SKBr3 cells were treated with DMSO (D), siramesine (S, 10 *μ*M), lapatinib (L, 0.5 *μ*M) or in combination (S+L) for 1, 2, 4, 6 and 24 h, respectively. Cell death was quantified as above. (**d**) Cell survival was test by using MTT assay following siramesine and lapatinib treatment. (**e**) Cells were then treated as before in the presence or absence of Ferrostatin-1 (5 *μ*M) and Necrostain-1 (50 *μ*M). Cell death was determined as above at 4 h. (**f**,**g**) MDA MB 231 cells were also treated as above in the presence or absence of DFO (0.1 mM). The cell death was determined at 4 h. Standard error represents three independent experiments (*n*=3). * represents statistical significance of *P*<0.05

To determine whether lysosomotropic agents are cytotoxic to breast cancer cells alone or in combination with chemotherapeutic agents, MDA MB-231 cells (triple negative breast cancer cell line) were treated with siramesine, cytotoxic agents (etoposide, cisplatin and taxol), anti-estrogen therapy (tamoxifan), and targeted chemotherapy, lapatinib (tyrosine kinase inhibitor against ErbB1/2), respectively. We found that the combination of siramesine (10 *μ*M) and lapatinib (0.5 *μ*M) gave a synergistic cell death response, whereas the combination of etoposide and siramesine gave an additive increase in cell death (Supplementary Figure 1). To confirm these results, we repeated these experiments in breast cancer cell lines MCF-7 and ZR-75-1 (estrogen receptor positive), and SKBr3 cells (estrogen receptor negative, ErbB2 over expressed) as these cell lines represent different subtypes of breast cancer. We found that the combination of siramesine and lapatinib-induced cell death to a great extent than the treatments alone, 10 *versus* 40% cell death (Figure 1a). The MDA MB 231 and SKBr3 cell lines were then treated over a 24 h time course and the amount of cell death determined. The combination of siramesine (10 *μ*M) and lapatinib (0.5 *μ*M) increased cell death in a time dependent manner with 35–45% cell death after 24 h. Treatment with DMSO, siramesine or lapatinib alone failed to induce cell death over this 24 h time course (Figures 1b and c). The MDA MB 231 and SkBr3 cell lines were also treated over a dose range of siramesine or lapatinib showing increased cell death in dose dependent manner (Supplementary Figure 2). Furthermore, using a cell viability assay (MTT assay), we showed decreased cell survival when siramesine and lapatinib were combined (Figure 1d). We also determine the extent of synergistic cell death using isobolograms. We found the combination index (CI) to be <1 for the combination of siramesine and lapatinib in both MDA MB 231 and SKBr3 cell lines indicating synergy (Supplementary Figure 3). We also determine whether this combination induces synergistic cell death in non-malignant cell lines. We treated the epithelial mammary cell line (MCF-10A1) with siramesine and lapatinib. We found the drug combination failed to significantly increased cell death compared to breast cancer cell lines (Supplementary Figure 5). These results suggest that siramesine and lapatinib-induced synergic cell death in breast cancer cells.

### Ferroptosis is induced by siramesine and lapatinib treatment

Lapatinib has been shown to induce apoptosis at high concentrations.^12, 13, 14^ We next determined whether combination of siramesine and lapatinib treatment-induced apoptosis. Treated cells were evaluated for apoptosis by using subG1 and Annexin V staining assays. We failed to observe significant apoptotic cell death after 4 h (Supplementary Figures 6A and B). To support this finding, we treated cells with caspase inhibitor z-VAD-fmk and determine the amount of total cell death. The caspase inhibitor failed to block siramesine and lapatinib-induced cell death (Supplementary Figure 6C) whereas z-VAD-fmk was effective at reducing cisplatin-induced cell death. (Supplementary Figure 6D).The caspase 3 activity was also unchanged after siramesine and lapatinib treatment (Supplementary Figure 6D). In addition, both PARP and caspase 3 failed to be cleaved following combined treatment with siramesine and lapatinib (Supplementary Figure 6E). These results suggest that the combination of siramesine and lapatinib is not inducing apoptosis. We next investigated whether cells were dying by necrosis or ferroptosis. We found that LDH release did not changed after siramesine and lapatinib treatment (Supplementary Figure 6F). We found that ferrostatin-1, which inhibits ferroptosis blocked siramesine and lapatinib-induced synergistic cell death whereas necrostain-1 that inhibits necrosis failed to protect against siramesine and lapatinib-induced cell death (Figure 1e). Similar results were observed in SKBr3 cells (Supplementary Figure 7). Ferroptosis is an iron-dependent type of cell death,^1^ and is defined by lipid peroxidation. Lipid ROS level was increased after siramesine and lapatinib treatment (Supplementary Figure 8). To identify the role of iron in siramesine and lapatinib-induced cell death, we treated cells with the iron chelator deferoxamine (DFO). This resulted in a decrease in siramesine and lapatinib-induced cell death from 54 to 35% in MDA MB 231 cells and from 64 to 42% in SkBr3 cells (Figures 1f and g). These results suggest that siramesine and lapatinib-induced ferroptotic cell death in breast cancer cells.

### ROS regulates siramesine and lapatinib-induced cell death

**Figure 2:**
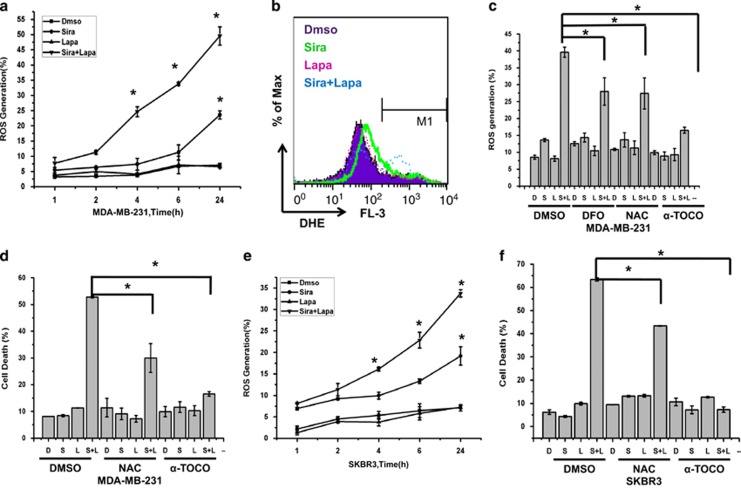
ROS regulates siramesine and lapatinib-induced cell death. (**a**) MDA MB 231 cells were treated with DMSO (D), siramesine (Sira, 10 *μ*M), lapatinib (Lapa, 0.5 *μ*M), or siramesine and lapatinib (Sira+Lapa) for 1, 2, 4, 6 and 24 h, respectively. ROS generation was quantified by flow cytometry as described in the Materials and Methods section. (**b**) ROS generation was demonstrated by flow cytometry with dihydroethidium (DHE, 10 *μ*M) after MDA MB 231 cells treated for 4 h. (**c**) Anti-oxidantsdesferoxamine (DFO, 0.1 mM), *N*-acetyl-l-cysteine (NAC, 1mM) and *α*-tocopherol (*α*-TOCO,10 *μ*M) were added in cells after treatment with DMSO (D), siramesine (S), lapatinib (L) and siramesine and lapatinib (S+L). The generation of ROS was determined as above. (**d**) These antioxidants in combination with treatments as described above were then analyzed for cell death with trypan blue exclusion assay. (**e**) SKBr3 cells were treated with DMSO, siramesine (Sira, 10 *μ*M), lapatinib (Lapa, 0.5 *μ*M), or siramesine and lapatinib (Sira and Lapa) for 1, 2, 4, 6 and 24 h, respectively. ROS generation was quantified by flow cytometry. (**f**) Antioxidants were combined with DMSO (D), siramesine (S), lapatinib (L) and siramesine and lapatinib (S+L) and cell death determined in SKBr3 cells as described above. Standard error represents three independent experiments (*n*=3). * represents statistical significance of *P*<0.05

Previous studies demonstrated that ferroptosis depends on ROS for its cytotoxicity.^1^ We then measure the amount of ROS generated over a 24 h time course in MDA MB 231 cells following treatment with siramesine and/or lapatinib. Similar to cell death, ROS generation was increased by 70 and 91% at 4 h and 24 h, respectively, compared to DMSO and lapatinib treated cells (Figures 2a and b). At 24 h, siramesine alone showed a 25% increase in ROS production, which has been previously demonstrated.^7^ We then inhibited ROS with antioxidants NAC and *α*-tocopherol and found they were both effective at blocking siramesine and lapatinib-induced ROS generation (Figure 2c). Treatment with the iron chelator DFO also effectively reduced ROS levels (Figure 2c). We also measured cell death following the addition of antioxidants NAC and *α*-tocopherol and found that siramesine and lapatinib-induced cell death was decreased by 40 and 66%, respectively, at 4 h (Figure 2d). Similarly in SKBr3 cells, ROS generation was increased by 53 and 78% at 4 h and 24 h, respectively, and cell death was inhibited by NAC, and *α*-tocopherol by 32 and 84%, respectively, following treatment with siramesine and lapatinib (Figures 2e and f). These results suggest that ROS have a role in siramesine and lapatinib-induced cell death.

### ROS generation is due in part to iron

ROS is generated from the lysosomes, mitochondria and membrane NADPH oxidases.^15, 16, 17^ To determine the cellular sources of ROS following siramesine and lapatinib treatment, intact mitochondria was quantified by flow cytometry using Mitotracker. There was no changed after treating with siramesine and lapatinib over 24 h time course in MDA MB 231 cells. Using a DIOC6 dye that detects mitochondrial membrane potential, there was also no change following siramesine and lapatinib treatment (Supplementary Figure 9A and B). To demonstrate whether ROS generation is from NADPH oxidases, ROS generation was measured after MDA MB 231 cells were pretreated with NADPH oxidase inhibitor DPI and Neopterin. These inhibitors failed to change ROS levels following siramesine and lapatinib treatment after 4 h (Supplementary Figure 9C). Lysosomes are another source of ROS due to the low pH and high iron content within the lysosome. Treatment of cells with DFO prevented siramesine and lapatinib-induced ROS generation (Figure 2c; Supplementary Figure 9C), indicating that the iron chelator was acting on the lysosomal production of ROS. We then measured intracellular iron by using Prussian blue cellular staining.^18^ Iron level significantly increased after treatment with siramesine and lapatinib (Supplementary Figure 9D). To further confirm that iron has a role in siramesine and lapatinib-induced cell death, exogenous Fe (FeCl_3_) was added, and the amount of cell death was further increased (Supplementary Figure 9E).These results suggest that ROS generation was due to iron and this has a critical role in siramesine and lapatinib-induced ferroptosis.

### Expression of iron regulatory proteins in MDA MB 231 and SKBr3 cells

**Figure 3:**
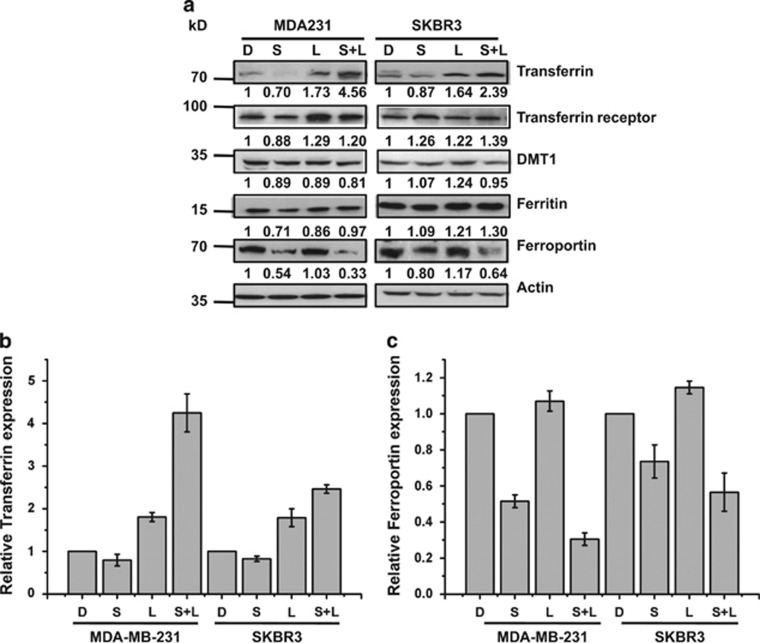
Expression of iron regulatory proteins in MDA MB 231 and SKBr3 cells. (**a**) MDA MB-231 and SKBr3 cells were lysed after treatment with DMSO (D), siramesine (S), lapatinib (L) and siramesine and lapatinib (S+L). Western blot determination of iron-related proteins transferrin, transferrin receptor, DMT1, ferritin, FPN was performed. Densitometry quantification of (**b**) transferrin and (**c**) FPN as normalized to actin, respectively

Iron levels are actively regulated in cells through transferrin that transports iron into cells and ferroportin that exports iron out of cells.^19, 20^ We investigated whether iron regulatory proteins such as transferrin and ferroportin are altered after siramesine and lapatinib treatment that might explain why iron is accumulating in cells. Cells were treated with siramesine and lapatinib for 4 h, and lysed and western blotted for transferrin and ferroportin protein. The expression of ferroportin (FPN) was significantly decreased after siramesine alone or the combination of siramesine and lapatinib. In contrast, the expression of transferrin significantly increased after lapatinib alone and in combination with siramesine and lapatinib in MDA MB 231 and SKBr3 cells (Figures 3a–c). To ensure this effect was not due to general reduction in iron transport regulatory proteins, we found that transferrin receptor, DMT1 and ferritin were not significantly changes following treatment (Figure 3a). These results indicate siramesine and lapatinib treatment alters iron transport in cells leading to cell death through ferroptosis.

### FPN and transferrin have a role in siramesine and lapatinib-induced cell death

**Figure 4:**
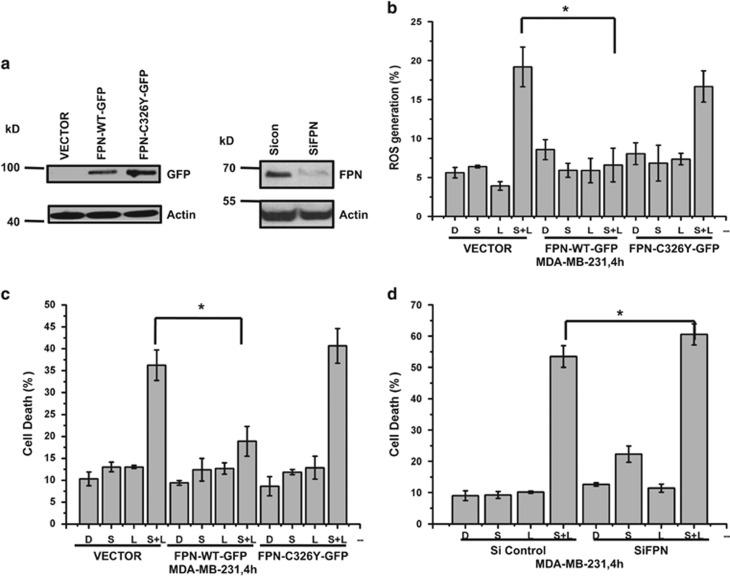

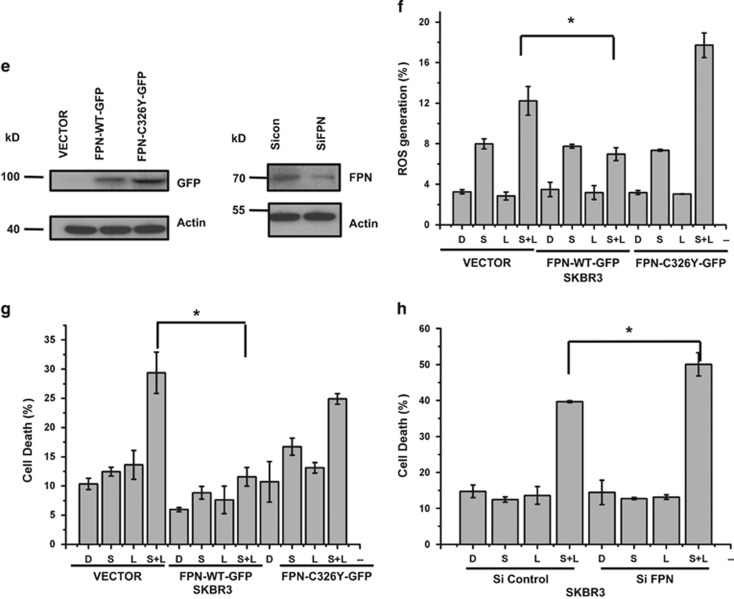
FPN overexpression alters siramesine and lapatinib-induced ferroptosis. (**a**) Overexpression of FPN by transfection of plasmids (Vector, FPN-WT-GFP, and FPN-C326Y-GFP) and knockdown of *fpn* by small interfering RNAs (siRNAs) was demonstrated by western blot of GFP and FPN, respectively, in MDA MB 231 cells. (**b**) Overexpression of *FPN* in MDA MB 231 cells were treated with DMSO (**d**), siramesine (S) and siramesine and lapatinib (S+L) and ROS generation measured at 4 h. (**c**) MDA MB 231 cells overexpressing *FPN* were treated as above and amount of cell death was determined by trypan blue exclusion assay at 4 h. (**d**) Knockdown of *FPN* in MDA MB 231 cells were also treated as above and amount of cell death at 4 h was determined. (**e**) Overexpression of FPN by transfection of plasmids (Vector, FPN-WT-GFP and FPN-C326Y-GFP) and knockdown of *fpn* by siRNAs was demonstrated by western blot of GFP and FPN, respectively, in SKBr3 cells. SKBr3 cells overexpressing *FPN* was treated as above and (**f**) amount of ROS determined by flow cytometry and (**g**) amount of cell death at 4 h determined. (**h**) Knockdown of *FPN* in SKBr3 cells were treated as above and amount of cell death determined after 4 h. Standard error represents three independent experiments (*n*=3). * represents statistical significance of *P*<0.05

To determine whether FPN expression can block siramesine and lapatinib-induced ROS generation and cell death, we transiently transfected cells with plasmid FPN-WT-GFP and FPN-C326Y-GFP (inactive form). When compared to vector and mutant FPN-C326Y-GFP, FPN-WT-GFP can decrease the ROS generation by 68% and cell death by 51% in MDA MB 231 cells. Conversely when we knocked down FPN, we observed an increase in cell death after siramesine and lapatinib treatment (Figures 4a–d). Similar results were observed in SKBr3 cells (Figures 4e–h). When we treated cells with erastin that induces ferroptosis, we found FPN overexpression failed to affect erastin-induced cell death (Supplementary Figure 10).

**Figure 5:**
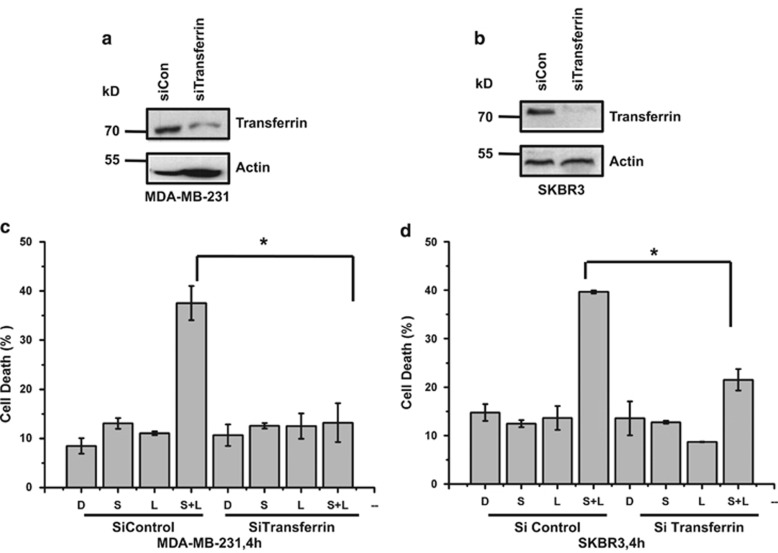
Silencing of transferrin expression by siRNA decreases cell death induced by siramesine and lapatinib. MDA MB 231 cells were knockdown in (**a**) transferrin receptor (TfR) and (**b**) transferrin expression by siRNAs as demonstrated by western blot. (**c**) Knocked down of transferrin (siTransferrin) was performed. Cells were treated with DMSO (**d**), siramesine(S), lapatinib (L) and siramesine and lapatinib (S+L). The amount of cell death after 4 h was determined by trypan blue exclusion assays. Cells transfected with siControl were used as a control. (**d**) Knocked down of transferrin (siTransferrin) in SKBr3 cells was performed and the cells were treated as described above. The amount of cell death was determined at 4 h. Standard error represents three independent experiments (*n*=3). * represents statistical significance of *P*<0.05

Lapatinib alone and in combination with siramesine increased the expression of transferrin (Figures 3a–c). To determine whether transferrin expression leads to siramesine and lapatinib-induced ROS and cell death. We knocked down the expression of transferrin, and compared it to the siRNA control in MDA MB 231 cells (Figures 5a and c). We found that knockdown of transferrin blocked siramesine and lapatinib-induced ROS and cell death. Similar results were observed in SKBr3 cells (Figures 5b and d). These results suggest that FPN and transferrin is involved in siramesine and lapatinib-induced ferroptotic cell death.

### Cystine transport inhibition promotes siramesine and lapatinib-induced cell death

**Figure 6:**
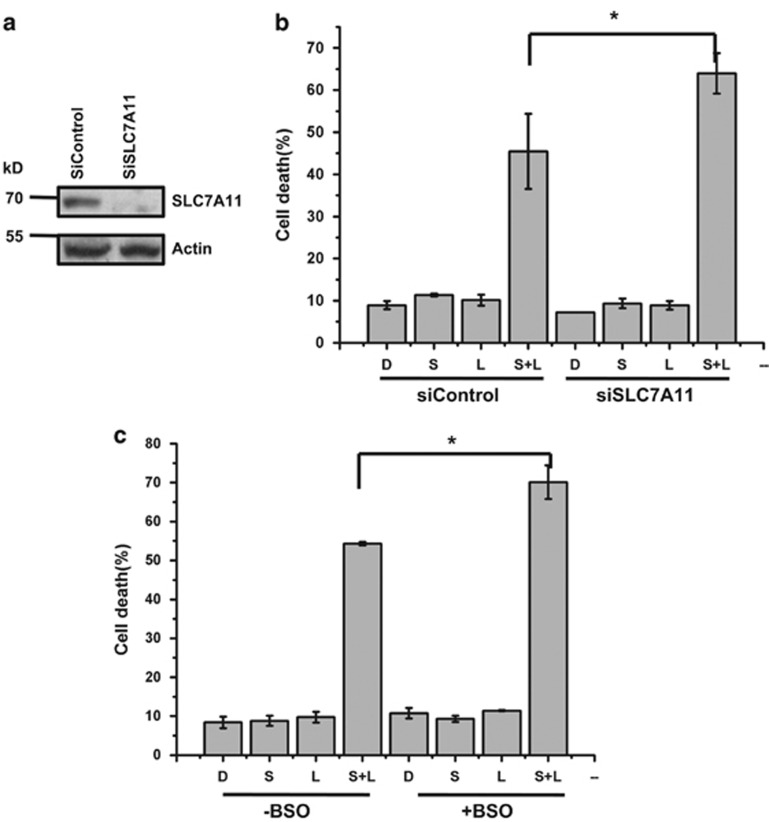
Cystine transport inhibition promotes siramesine and lapatinib-induced cell death. (**a**) Knockdown of SLC7A11 by siRNAs (siSLC7A11) in MDA MB 231 cells was determined by western blotting of SLC7A11. (**b**) MDA MB 231 cells knocked down for SLC7A11 expression were treated with DMSO (D), siramesine (S), lapatinib (L) and siramesine and lapatinib (S+L). The amount of cell death was determined at 4 h. (**c**) MDA MB 231 cells were treated as above in the presence and absence BSO (1mM), pretreated for 1 h. The amount of cell death was determined using trypan blue exclusion assay at 4 h. Standard error represents three independent experiments (*n*=3). *represents statistical significance of *P*<0.05

Inhibition of the cystine transport receptor, XCT (SLC7A11) contributes to ferroptosis.^1, 21, 22^ We knocked down SLC7A11 (Figure 6a) and found that siramesine and lapatinib-induced cell death was significantly increased (Figure 6b). This was similar to increased cell death induced by erastin treatment following SLC7A11 knockdown in cells (Supplementary Figure 11). We also found that SLC7A11 protein levels increased following siramesine and lapatinib treatment or erastin treatment (Supplementary Figure 12). Glutathione syntheses (GSH) is activated downstream of SLC7A11 contributing to ferroptosis. We then depleted GSH in cells by treating with buthioninesulphoximine (BSO) and found that siramesine and lapatinib-induced cell death was increased (Figure 6c). This was similar to erastin treatment where GSH depletion increased erastin-induced cell death (Supplementary Figure 13). This indicates the cystine regulation promotes ferroptosis following siramesine and lapatinib treatment similar to erastin.

Ferroptosis is also regulated by glutathione peroxidase 4 (Gpx4).^23^ We found that knocking down GPX4 increased siramesine and lapatinib or erastin-induced cell death in both MDA MB 231 and SKBr3 cells. This is consistent with siramesine and lapatinib-inducing ferroptosis. (Supplementary Figure 14).

### Downstream targets for lapatinib and siramesine are not associated with the synergistic cell death response

Lapatinib effectively inhibits the epidermal growth factor receptors, ErbB1 (EGFR) and ErbB2 (HER2) in breast cancer cells.^24, 25^ To determine whether these receptors have a role in siramesine and lapatinib-induced cells death, we knocked down HER2 alone, EGFR alone, and then EGFR and HER2 together through transiently using siRNA in MDA MB 231 cells. We found that knocking down these proteins had no effect on siramesine and lapatinib-induced cell death. There was no difference in the amount of cell death between siRNA control and knockdown cell lines following 4 h of treatment (Supplementary Figure 15). Furthermore, we test another EGFR inhibitor, gefitinib, which also had no synergistic effect on cell death when combined with siramesine (Supplementary Figure 16). This indicates that lapatinib combined with siramesine induces cell death independent of EGFR or HER2.

Siramesine can induce lysosomal membrane permeabilization (LMP) leading to cathepsin B mediated cell death.^26, 27^ We determine the amount of LMP by flow cytometry using AO staining. LMP occurred after siramesine alone, but the combination of lapatinib and siramesine failed to further increase LMP. Pre-treatment with DFO reduced the amount of LMP seen in cells that were treated with both siramesine and lapatinib (Supplementary Figure 17). We then treated cells with cathepsin B inhibitors CA-074Me and determine the amount of cell death. We found that the inhibitor fails to prevent siramesine and lapatinib-induced cell death (Supplementary Figure 18). This suggests that the combination of lapatinib and siramesine act independently of cathepsin B for their synergistic cell death response.

Both sirasesine and laptinib induce autophagy.^28, 29^ Autophagy has been shown to contribute to both cell survival and cell death.^30^ To determine whether autophagy is involved in siramesine and lapatinib-induced cell death. We have treated cells autophagy inhibitors 3-MA and spautin-1. We found that 3-MA and spautin-1 increased siramesine and lapatinib-induced cell death suggesting autophagy contributes to cell survival. (Supplementary Figure 19).

## Discussion

Ferroptosis is dependent upon intracellular iron, but not other metals, and is morphologically, biochemically and genetically distinct from apoptosis, necrosis and autophagy.^1, 22, 31, 32, 33, 34, 35, 36, 37^ There are few inducers of ferroptosis that have been identified. Erastin, sulfasalazine, RSL3 and cysteine starvation are the most often investigated.^15, 21^ We found that the combination of lysosomotropic agent siramesine, that has been shown to be effective in inducing cell death in breast cancer cells, and a kinase inhibitor lapatinib, that is used in the treatment of breast cancer, induced ferroptosis. This combination induced ferroptosis through decreased ferroportin expression and increased transferrin expression leading to increased iron. Iron catalyzes Fenton reactions, yielding extremely reactive hydroxyl radicals, enhanced ROS generation resulting in cell death (Supplementary Figure 20).

Proteins involved in iron transport, including transferrin and FPN. Ferroportin, the major iron efflux transporter in the cell plasma membrane, which will release Fe^2+^ from cells.^21^ Ferroportin is also a critical control site for recycling iron according to need; its expression levels on the plasma membrane being controlled mainly by the small peptide hepcidin. Knocking out ferroportin expression results in a profound iron deficiency ascribable not only to reduced absorption of dietary iron, but also to a reduction in cycling of iron in red cell hemoglobin.^20, 38^ Indeed, knockout mice lacking ferroportin are embryonic lethal. However in breast cancer cells, down regulation of ferroportin promotes breast cancer growth and is correlated with poor prognosis.^39^ This suggests iron regulation is altered in breast cancer and could be a target for cancer therapy.

Transferrin is another iron-transport protein. Transferrin receptor 1 mediates most of the cellular iron uptake by binding iron-transferrin at cell surface, which is internalized by receptor-mediated endocytosis; release and reduction of the iron in endosomes and transport of the released iron into the cytosol.^40, 41^ Recently, under starvation conditions, it was shown transferrin import is required for ferroptosis and could be important in ischemia/reperfusion induced heart injury.^42^ Erastin-induced cell death was however independent of ferroportin and transferrin in breast cancer cells. This suggests that siramesine and lapatinib-induced ferroptosis depends on a novel regulation of iron transport in breast cancer cells.

One of the keys in regulating ferroptosis is the generation of ROS. Ferroptosis is prevented by lipophilic antioxidants, such as trolox and vitamin E, and by iron chelators such as deferoxamine but not by well-known small-molecule inhibitors of apoptosis, necrosis or autophagy.^2, 43^ Our results agree with this prevention of ferroptosis. Inhibition of heme-dependent NADPH oxidase enzymes is also able to prevent ferroptosis in some cell types but was not responsible for ROS generated following siramesine and lapatinib treatment. Cystine transport through the cystine/glutamate antiporter (system *x*_c_^−^) is involved in regulating ROS through activation of GSH.^44, 45, 46^ Glutathione depletion leads to the iron-dependent accumulation of ROS, especially lipid ROS.^47, 48^ The cystine uptake inhibitor erastin has been shown to block GSH activation leading to accumulation of ROS and ferroptosis.^23, 33^ We found that both erastin and the combination of siramesine and lapatinib-induced cell death was promoted by inhibition of cystine transport. Overall, our results provides evidence that combining siramesine and lapatinib causes ferroptosis through iron transport disruption leading to increased ROS. This is promoted by altering cystine transport.

Lapatinib was developed as a dual kinase inhibitor of EGFR and HER2 and siramesine disrupts lysosomes leading to cathepsin-mediated cell death.^3^ We found that synergistic cell death response of combining siramesine and lapatinib leading to ferroptosis was not due to their downstream targets (EGFR family members) and cathepsin B. This indicates other targets for siramesine and lapatinib are involved in ferroptosis. These drugs have multiple targets including ASM and src family of kinases.^49, 50^ Indeed, a src family tyrosine kinase inhibitor, sorafenib has been shown to induce ferroptosis in cancer cells.^21, 51^ Identification of these targets and how they regulate ferroptosis will be the focus of future investigations.

Taken together, our findings provide a novel mechanism for ferroptosis that involves altering iron transport in breast cancer cells. Using clinically relevant drugs, these results provide new insight into how cancer cells are able to induce the ferroptotic cell death process and give hope that new therapeutic strategies can be develop to overcome apoptotic resistance in breast cancer.

## Materials and Methods

### Reagents and antibodies

Trypan blue solution (Prod. No. T8154), Prussian blue soluble (Prod No.03899), FeCl_3_ (Prod No.157740), deferoxamine (Prod No.D9533), buthioninesulfoximine (BSO) (Prod No.B2640), *N*-Acetyl-l-cysteine (Prod No. N7250), diphenyliodonium (Prod No. D2926), neopterin (Prod No. N3386), and phosphatase inhibitor cocktails 2 & 3 (Prod No. P5726 & P0044) were purchased from Sigma-Aldrich (St. Louis, MO, USA), A protease inhibitor cocktail (ref no. 11 836 153 001) from Roche Diagnostics (Basel, Switzerland). The siRNA against HER2 (sc-29405), EGFR (sc-44340), transferrin (sc-37176), ferroportin (sc-60663), XCT (sc-76933) and Control siRNA-A (Sc-37007) were all purchased from Santa Cruz Biotechnology Inc. (Dallas, TX , USA). Primary antibodies: anti-FTH1 (#4393), anti-CD71 (#13113), anti-xCT/SLC7A11 (#12691) were purchased from Cell Signaling Technology (Beverly, MA, USA), anti-Transferrin (ab9538), anti-DMT1 (ab55735), anti-SLC40A1 (ab85370), anti-GPX4 (ab125066) from AbCam (Cambridge, UK), and anti-actin from Sigma-Aldrich (Prod No. A3853). Secondary antibodies: goat anti-rabbit IgG (H+L)-HRP conjugate (Cat. No. 170-6515) and goat anti-mouse IgG (H+L)-HRP conjugate (Cat. No. 170-6516) were obtained from Bio-Rad Laboratories (Hercules, CA, USA) (ab9538). FPN-WT-GFP and FPN-C326Y-GFP plasmid kindly provided by Dr. Sergei Nekhai. HER2-WT (Plasmid #16257),was obtained from Addgene (Cambridge, MA, USA). MitoTracker (Catalog number: M-7512), LysoTracker (cat: L-7526), H2DCF (cat: D-399), Dihydroethidium (Cat: D-1168) from Life Technologies (Thermo Fisher Scientific, Waltham, MA, USA). FITC Annexin V Apoptosis Detection Kit with 7AAD from BioLegend (San Diego, CA, USA). Opti-MEM I reduced serum medium (cat:31985-070) from GIBCO-Life Technologies (Thermo Fisher Scientific).

### Cell culture

The breast cancer cell lines MDA MB-231, SKBR3, MCF-7, ZR-75-1 were grown in Dulbecco's modified Eagle medium (DMEM, high glucose; GIBCO, cat. 10565-018, Life Technologies) supplemented with 100 units of penicillin per ml plus 100 *μ*g of streptomycin per ml (cat 10378016, Life Technologies) and 10% fetal bovine serum, in a humidified 5% CO_2_, 37 °C incubator. Cells were treated for various times in the absence and presence of a chemical inhibitor.

### Measurement of cell death by flow cytometry

Cell death was measured by staining with trypan blue to detect the plasma membrane integrity through flow cytometry as described previously.^30, 52^ Briefly, trypan blue is excluded from live cells but penetrates into a dead cell giving a red fluorescent signal that can be quantified by flow cytometry.

### Measurement of ROS by flow cytometry

ROS generation was determined by flow cytometry with dihydroethidium (DHE,D-1168). DHE is oxidized by ROS into 2-hydroxyethidium (2-HE) (emission at 605 nm) and fluoresces red. The samples were collected and stained with 5 *μ*M DHE and then was incubated in the dark in a water bath at 37 °C for 15 min. The cell suspension was then transferred to a 5 ml FACS tube and analyzed on a flow cytometer within 10 min using Cell Quest software (BD Biosciences, Franklin Lakes, NJ, USA).

### Apoptotic cell death measurement

Flow cytometric analysis of apoptosis was analyzed using both subG1 peak (DNA fragmentation) and Annexin V/7-AAD which measures the change in both membrane phospholipid phosphatidylserine and cell permeabilization. Cells were stained for subG1 peak analysis after fixation with ethanol using propidium iodide (100 *μ*g/ml). For Annexin V/7-AAD, samples were collected, washed with 11 × annexin V binding buffer (BD Biosciences), and then stained with 7AAD (BD) and annexin V-fluorescein isothiocyanate (FITC; BD). Samples were examined using a BD FACSCalibur.

### Transfection of siRNA and plasmids

The transfection of cells with siRNA and plasmid was done as described in our previously studies.^30, 52^

### Flow cytometry for mitochondrial membrane permeabilization (MMP), mitochondrial membrane potential (Δ*ψ*m) and LMP detection

The MMP and mitochondrial membrane potential (Δ*ψ*m) of the treated cells was measured using MitoTracker Deep Red FM and DIOC6 staining. In brief, the culture medium was aspirated after treatment, and the cells were collected and incubated with MitoTracker Deep Red FM, 100 nM in PBS, Dioc6,1 *μ*M in PBS, LysoTracker 50 nM for 15 min at 37 °C. The fluorescence emission was analyzed by flow cytometry using a FACS Vantage system (Becton Dickinson Inc., San Jose, CA, USA).

### Western blot analysis

Cell lysates were collected at the indicated times in 1% NP-40 lysis buffer with complete protease inhibitor tablet (Roche, Basel, Switzerland), 1 mM phenylmethanesulfonylfluoride (PMSF), and 2 mM sodium orthovanadate (New England BioLabs, Ipswich, MA, USA). Protein levels were quantified with a Pierce BCA kit (Thermo Fisher Scientific) according to the manufacturer's instructions. Samples were run on 8–10% polyacrylamide gels and transferred onto nitrocellulose membranes (Bio-Rad, Hercules, CA, USA) blocked in 5% milk in TBS-T as per the antibody manufacturer's suggestions. Secondary antibodies were goat anti-rabbit-HRP or anti-mouse-HRP (Bio-Rad). Detection of protein was with Pierce ECL or Pierce Supersignal Pico (Thermo Fisher Scientific) reagents.

### Prussian blue staining

Prussian blue staining was used to detect the presence of iron oxide nanoparticles. The cells were fixed in 4% paraformaldehyde for 30 min and then were washed 3 times with PBS, incubated for 30 min with Prussian blue (10 mg/ml), and then rewashed three times with PBS. Labeled cells were examined under a light microscope to determine intracellular iron oxide distribution.

### Caspase-3 activity assay

Caspase-3 activity was assessed using a caspase-3 assay Kit (#ab 39700, Abcam) according to the manufacturer's instructions.

### LDH release assay

LDH release was assessed using a LDH assay kit (#ab 65393, Abcam) according to the manufacturer's instructions.

### Assessment of drug interaction

Interaction between siramesine and lapatinib was assessed according to the method of Chou and Talalay,^53^ where the Combination Index (CI) is defined as:


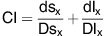


Where ds_x_ and dl_x_ are doses of siramesine and lapatinib, respectively, required to produce a given reduction in cell viability when given in combination, and Ds*_x_* and Dl*_x_* are doses of siramesine and lapatinib, respectively, required to produce the same effect in single-agent treatments. CI<1, =1 and >1 are interpreted as synergy, additivity and antagonism, respectively.

### Statistical analysis

All data were generated with at least three independent experiments. Each experiment in the cell death analysis was carried out by 3–6 replicates. The data were represented as means ± S.D. (*n*≥3). Student's *t*-test was performed for statistical analysis with *P*<0.05 being considered as statistical significance.

## Abbreviations

2-HE: 2-hydroxyethidium
ASM: acid sphingomyelinase
BSO: buthioninesulphoximine
CI: Combination Index
DHE: dihydroethidium
EGFR: epidermal growth factor receptor
DFO: deferoxamine
D: DMSO
GSH: glutathione syntheses (GSH)
GPX4: glutathione peroxidase 4
FITC: fluorescein isothiocyanate
FPN: ferroportin-1
L: lapatinib
Lapa: lapatinib
LDH: lactate dehydrogenase
LMP: lysosomal membrane permeabilization
MMP: mitochondrial membrane permeabilization
Δ*ψ*m: mitochondrial membrane potential
NAC: *N*-acetyl-l-cysteine
PMSF: phenylmethanesulfonylfluoride
ROS: reactive oxygen species
S.D.: standard deviation
S: siramesine
Sira: siramesine
S+L: siramesine and lapatinib
Sira+Lapa: siramesine and lapatinib
siRNA: small interfering RNA
*α*-TOCO: *α*-tocopherol
TfR: transferrin receptor

## References

[bib1] Dixon SJ, Lemberg KM, Lamprecht MR, Skouta R, Zaitsev EM, Gleason CE et al. Ferroptosis: an iron-dependent form of nonapoptotic cell death. Cell 2012; 149: 1060–1072. 22632970 10.1016/j.cell.2012.03.042 PMC3367386

[bib2] Yagoda N, von Rechenberg M, Zaganjor E, Bauer AJ, Yang WS, Fridman DJ et al. RAS-RAF-MEK-dependent oxidative cell death involving voltage-dependent anion channels. Nature 2007; 447: 864–868. 17568748 10.1038/nature05859 PMC3047570

[bib3] Christofferson DE, Yuan J. Necroptosis as an alternative form of programmed cell death. Curr Opin Cell Biol 2010; 22: 263–268. 20045303 10.1016/j.ceb.2009.12.003 PMC2854308

[bib4] Jacobson MD, Raff MC. Programmed cell death and Bcl-2 protection in very low oxygen. Nature 1995; 374: 814–816. 7536895 10.1038/374814a0

[bib5] Boya P, Kroemer G. Lysosomal membrane permeabilization in cell death. Oncogene 2008; 27: 6434–6451. 18955971 10.1038/onc.2008.310

[bib6] Heading C. Siramesine H Lundbeck. Curr Opin Investig Drugs 2001; 2: 266–270. 11816842

[bib7] Ostenfeld MS, Fehrenbacher N, Hoyer-Hansen M, Thomsen C, Farkas T, Jaattela M. Effective tumor cell death by sigma-2 receptor ligand siramesine involves lysosomal leakage and oxidative stress. Cancer Res 2005; 65: 8975–8983. 16204071 10.1158/0008-5472.CAN-05-0269

[bib8] Rusnak DW, Lackey K, Affleck K, Wood ER, Alligood KJ, Rhodes N et al. The effects of the novel, reversible epidermal growth factor receptor/ErbB-2 tyrosine kinase inhibitor, GW2016, on the growth of human normal and tumor-derived cell lines *in vitro* and *in vivo*. Mol Cancer Ther 2001; 1: 85–94. 12467226

[bib9] Wood ER, Truesdale AT, McDonald OB, Yuan D, Hassell A, Dickerson SH et al. A unique structure for epidermal growth factor receptor bound to GW572016 (Lapatinib): relationships among protein conformation, inhibitor off-rate, and receptor activity in tumor cells. Cancer Res 2004; 64: 6652–6659. 15374980 10.1158/0008-5472.CAN-04-1168

[bib10] Xia J, Deng H, Feng Y, Zhang H, Pan Q, Dai H et al. A novel locus for autosomal dominant nonsyndromic hearing loss identified at 5q31.1-32 in a Chinese pedigree. J Hum Genet 2002; 47: 635–640. 12522684 10.1007/s100380200098

[bib11] Wetterskog D, Shiu KK, Chong I, Meijer T, Mackay A, Lambros M et al. Identification of novel determinants of resistance to lapatinib in ERBB2-amplified cancers. Oncogene 2014; 33: 966–976. 23474757 10.1038/onc.2013.41

[bib12] Park SH, Ito K, Olcott W, Katsyv I, Halstead-Nussloch G, Irie HY. PTK6 inhibition promotes apoptosis of Lapatinib-resistant Her2(+) breast cancer cells by inducing Bim. Breast Cancer Res 2015; 17: 86. 26084280 10.1186/s13058-015-0594-z PMC4496943

[bib13] Nahta R, Yuan LX, Du Y, Esteva FJ. Lapatinib induces apoptosis in trastuzumab-resistant breast cancer cells: effects on insulin-like growth factor I signaling. Mol Cancer Ther 2007; 6: 667–674. 17308062 10.1158/1535-7163.MCT-06-0423

[bib14] Tanizaki J, Okamoto I, Fumita S, Okamoto W, Nishio K, Nakagawa K. Roles of BIM induction and survivin downregulation in lapatinib-induced apoptosis in breast cancer cells with HER2 amplification. Oncogene 2011; 30: 4097–4106. 21499301 10.1038/onc.2011.111

[bib15] Dixon SJ, Stockwell BR. The role of iron and reactive oxygen species in cell death. Nat Chem Biol 2014; 10: 9–17. 24346035 10.1038/nchembio.1416

[bib16] Gorlach A, Dimova EY, Petry A, Martinez-Ruiz A, Hernansanz-Agustin P, Rolo AP et al. Reactive oxygen species, nutrition, hypoxia and diseases: Problems solved? Redox Biol 2015; 6: 372–385. 26339717 10.1016/j.redox.2015.08.016 PMC4565025

[bib17] Heid ME, Keyel PA, Kamga C, Shiva S, Watkins SC, Salter RD. Mitochondrial reactive oxygen species induces NLRP3-dependent lysosomal damage and inflammasome activation. J Immunol 2013; 191: 5230–5238. 24089192 10.4049/jimmunol.1301490 PMC3833073

[bib18] Shahbazi-Gahrouei D, Abdolahi M. Superparamagnetic iron oxide-C595: Potential MR imaging contrast agents for ovarian cancer detection. J Med Phys 2013; 38: 198–204. 24672155 10.4103/0971-6203.121198 PMC3959000

[bib19] Gkouvatsos K, Papanikolaou G, Pantopoulos K. Regulation of iron transport and the role of transferrin. Biochim Biophys Acta 2012; 1820: 188–202. 22085723 10.1016/j.bbagen.2011.10.013

[bib20] Ward DM, Kaplan J. Ferroportin-mediated iron transport: expression and regulation. Biochim Biophys Acta 2012; 1823: 1426–1433. 22440327 10.1016/j.bbamcr.2012.03.004 PMC3718258

[bib21] Dixon SJ, Patel DN, Welsch M, Skouta R, Lee ED, Hayano M et al. Pharmacological inhibition of cystine-glutamate exchange induces endoplasmic reticulum stress and ferroptosis. Elife 2014; 3: e02523. 24844246 10.7554/eLife.02523 PMC4054777

[bib22] Jiang L, Kon N, Li T, Wang SJ, Su T, Hibshoosh H et al. Ferroptosis as a p53-mediated activity during tumour suppression. Nature 2015; 520: 57–62. 25799988 10.1038/nature14344 PMC4455927

[bib23] Yang WS, SriRamaratnam R, Welsch ME, Shimada K, Skouta R, Viswanathan VS et al. Regulation of ferroptotic cancer cell death by GPX4. Cell 2014; 156: 317–331. 24439385 10.1016/j.cell.2013.12.010 PMC4076414

[bib24] Tebbutt N, Pedersen MW, Johns TG. Targeting the ERBB family in cancer: couples therapy. Nat Rev Cancer 2013; 13: 663–673. 23949426 10.1038/nrc3559

[bib25] Larbouret C, Gaborit N, Chardes T, Coelho M, Campigna E, Bascoul-Mollevi C et al. In pancreatic carcinoma, dual EGFR/HER2 targeting with cetuximab/trastuzumab is more effective than treatment with trastuzumab/erlotinib or lapatinib alone: implication of receptors' down-regulation and dimers' disruption. Neoplasia 2012; 14: 121–130. 22431920 10.1593/neo.111602 PMC3306257

[bib26] Johansson AC, Appelqvist H, Nilsson C, Kagedal K, Roberg K, Ollinger K. Regulation of apoptosis-associated lysosomal membrane permeabilization. Apoptosis 2010; 15: 527–540. 20077016 10.1007/s10495-009-0452-5 PMC2850995

[bib27] Ghosh M, Carlsson F, Laskar A, Yuan XM, Li W. Lysosomal membrane permeabilization causes oxidative stress and ferritin induction in macrophages. FEBS Lett 2011; 585: 623–629. 21219901 10.1016/j.febslet.2010.12.043

[bib28] Ostenfeld MS, Hoyer-Hansen M, Bastholm L, Fehrenbacher N, Olsen OD, Groth-Pedersen L et al. Anti-cancer agent siramesine is a lysosomotropic detergent that induces cytoprotective autophagosome accumulation. Autophagy 2008; 4: 487–499. 18305408 10.4161/auto.5774

[bib29] Zambrano J, Yeh ES. Autophagy and apoptotic crosstalk: mechanism of therapeutic resistance in HER2-positive breast cancer. Breast Cancer (Auckl) 2016; 10: 13–23. 26997868 10.4137/BCBCR.S32791 PMC4790584

[bib30] Maycotte P, Thorburn A. Targeting autophagy in breast cancer. World J Clin Oncol 2014; 5: 224–240. 25114840 10.5306/wjco.v5.i3.224 PMC4127596

[bib31] Friedmann Angeli JP, Schneider M, Proneth B, Tyurina YY, Tyurin VA, Hammond VJ et al. Inactivation of the ferroptosis regulator Gpx4 triggers acute renal failure in mice. Nat Cell Biol 2014; 16: 1180–1191. 25402683 10.1038/ncb3064 PMC4894846

[bib32] Wu D, Chen L. Ferroptosis: a novel cell death form will be a promising therapy target for diseases. Acta Biochim Biophys Sin (Shanghai) 2015; 47: 857–859. 26350095 10.1093/abbs/gmv086

[bib33] Matsushita M, Freigang S, Schneider C, Conrad M, Bornkamm GW, Kopf M. T cell lipid peroxidation induces ferroptosis and prevents immunity to infection. J Exp Med 2015; 212: 555–568. 25824823 10.1084/jem.20140857 PMC4387287

[bib34] Chen L, Hambright WS, Na R, Ran Q. Ablation of ferroptosis inhibitor glutathione peroxidase 4 in neurons results in rapid motor neuron degeneration and paralysis. J Biol Chem 2015; 290: 28097–28106. 26400084 10.1074/jbc.M115.680090 PMC4653669

[bib35] Linkermann A, Skouta R, Himmerkus N, Mulay SR, Dewitz C, De Zen F et al. Synchronized renal tubular cell death involves ferroptosis. Proc Natl Acad Sci U S A 2014; 111: 16836–16841. 25385600 10.1073/pnas.1415518111 PMC4250130

[bib36] Sun X, Ou Z, Chen R, Niu X, Chen, Kang R et al. Activation of the p62-Keap1-NRF2 pathway protects against ferroptosis in hepatocellular carcinoma cells. Hepatology 2015; 63: 173–184. 26403645 10.1002/hep.28251 PMC4688087

[bib37] Lorincz T, Jemnitz K, Kardon T, Mandl J, Szarka A. Ferroptosis is involved in acetaminophen induced cell death. Pathol Oncol Res 2015; 21: 1115–1121. 25962350 10.1007/s12253-015-9946-3

[bib38] Zhang Z, Zhang F, An P, Guo X, Shen Y, Tao Y et al. Ferroportin1 deficiency in mouse macrophages impairs iron homeostasis and inflammatory responses. Blood 2011; 118: 1912–1922. 21705499 10.1182/blood-2011-01-330324

[bib39] Miller LD, Coffman LG, Chou JW, Black MA, Bergh J, D'Agostino RJr. et al. An iron regulatory gene signature predicts outcome in breast cancer. Cancer Res 2011; 71: 6728–6737. 21875943 10.1158/0008-5472.CAN-11-1870 PMC3206152

[bib40] Chitambar CR, Wereley JP. Transferrin receptor-dependent and -independent iron transport in gallium-resistant human lymphoid leukemic cells. Blood 1998; 91: 4686–4693. 9616166

[bib41] Yamanishi H, Iyama S, Yamaguchi Y, Kanakura Y, Iwatani Y. Total iron-binding capacity calculated from serum transferrin concentration or serum iron concentration and unsaturated iron-binding capacity. Clin Chem 2003; 49: 175–178. 12507977 10.1373/49.1.175

[bib42] Gao M, Monian P, Quadri N, Ramasamy R, Jiang X. Glutaminolysis and transferrin regulate ferroptosis. Mol Cell 2015; 59: 298–308. 26166707 10.1016/j.molcel.2015.06.011 PMC4506736

[bib43] Yang WS, Stockwell BR. Synthetic lethal screening identifies compounds activating iron-dependent, nonapoptotic cell death in oncogenic-RAS-harboring cancer cells. Chem Biol 2008; 15: 234–245. 18355723 10.1016/j.chembiol.2008.02.010 PMC2683762

[bib44] Ishimoto T, Nagano O, Yae T, Tamada M, Motohara T, Oshima H et al. CD44 variant regulates redox status in cancer cells by stabilizing the xCT subunit of system xc(-) and thereby promotes tumor growth. Cancer Cell 2011; 19: 387–400. 21397861 10.1016/j.ccr.2011.01.038

[bib45] Lewerenz J, Hewett SJ, Huang Y, Lambros M, Gout PW, Kalivas PW et al. The cystine/glutamate antiporter system x(c)(-) in health and disease: from molecular mechanisms to novel therapeutic opportunities. Antioxid Redox Signal 2013; 18: 522–555. 22667998 10.1089/ars.2011.4391 PMC3545354

[bib46] Habib E, Linher-Melville K, Lin HX, Singh G. Expression of xCT and activity of system *x* are regulated by NRF2 in human breast cancer cells in response to oxidative stress. Redox Biol 2015; 5: 33–42. 25827424 10.1016/j.redox.2015.03.003 PMC4392061

[bib47] Chiste RC, Freitas M, Mercadante AZ, Fernandes E. Carotenoids inhibit lipid peroxidation and hemoglobin oxidation, but not the depletion of glutathione induced by ROS in human erythrocytes. Life Sci 2014; 99: 52–60. 24486304 10.1016/j.lfs.2014.01.059

[bib48] Skouta R, Dixon SJ, Wang J, Dunn DE, Orman M, Shimada K et al. Ferrostatins inhibit oxidative lipid damage and cell death in diverse disease models. J Am Chem Soc 2014; 136: 4551–4556. 24592866 10.1021/ja411006a PMC3985476

[bib49] Petersen NH, Olsen OD, Groth-Pedersen L, Ellegaard AM, Bilgin M, Redmer S et al. Transformation-associated changes in sphingolipid metabolism sensitize cells to lysosomal cell death induced by inhibitors of acid sphingomyelinase. Cancer Cell 2013; 24: 379–393. 24029234 10.1016/j.ccr.2013.08.003

[bib50] Zhang D, Pal A, Bornmann WG, Yamasaki F, Esteva FJ, Hortobagyi GN et al. Activity of lapatinib is independent of EGFR expression level in HER2-overexpressing breast cancer cells. Mol Cancer Ther 2008; 7: 1846–1850. 18644997 10.1158/1535-7163.MCT-08-0168 PMC2525738

[bib51] Lachaier E, Louandre C, Godin C, Saidak Z, Baert M, Diouf M et al. Sorafenib induces ferroptosis in human cancer cell lines originating from different solid tumors. Anticancer Res 2014; 34: 6417–6422. 25368241

[bib52] Azad MB, Chen Y, Henson ES, Cizeau J, McMillan-Ward E, Israels SJ et al. Hypoxia induces autophagic cell death in apoptosis-competent cells through a mechanism involving BNIP3. Autophagy 2008; 4: 195–204. 18059169 10.4161/auto.5278 PMC3164855

[bib53] Chou TC, Talalay P. Quantitative analysis of dose-effect relationships: the combined effects of multiple drugs or enzyme inhibitors. Adv Enzyme Regul 1984; 22: 27–55. 6382953 10.1016/0065-2571(84)90007-4

