# Supplementary material for: Ferroptosis is induced following siramesine and lapatinib treatment of breast cancer cells
Source: Cell Death Dis. 2016 Jul 21;7(7):e2307–. doi: 10.1038/cddis.2016.208 (PMC4973350; doi:10.1038/cddis.2016.208)
Supplement: Supplementary Figures [file cddis2016208x1.ppt]

## Slide 1
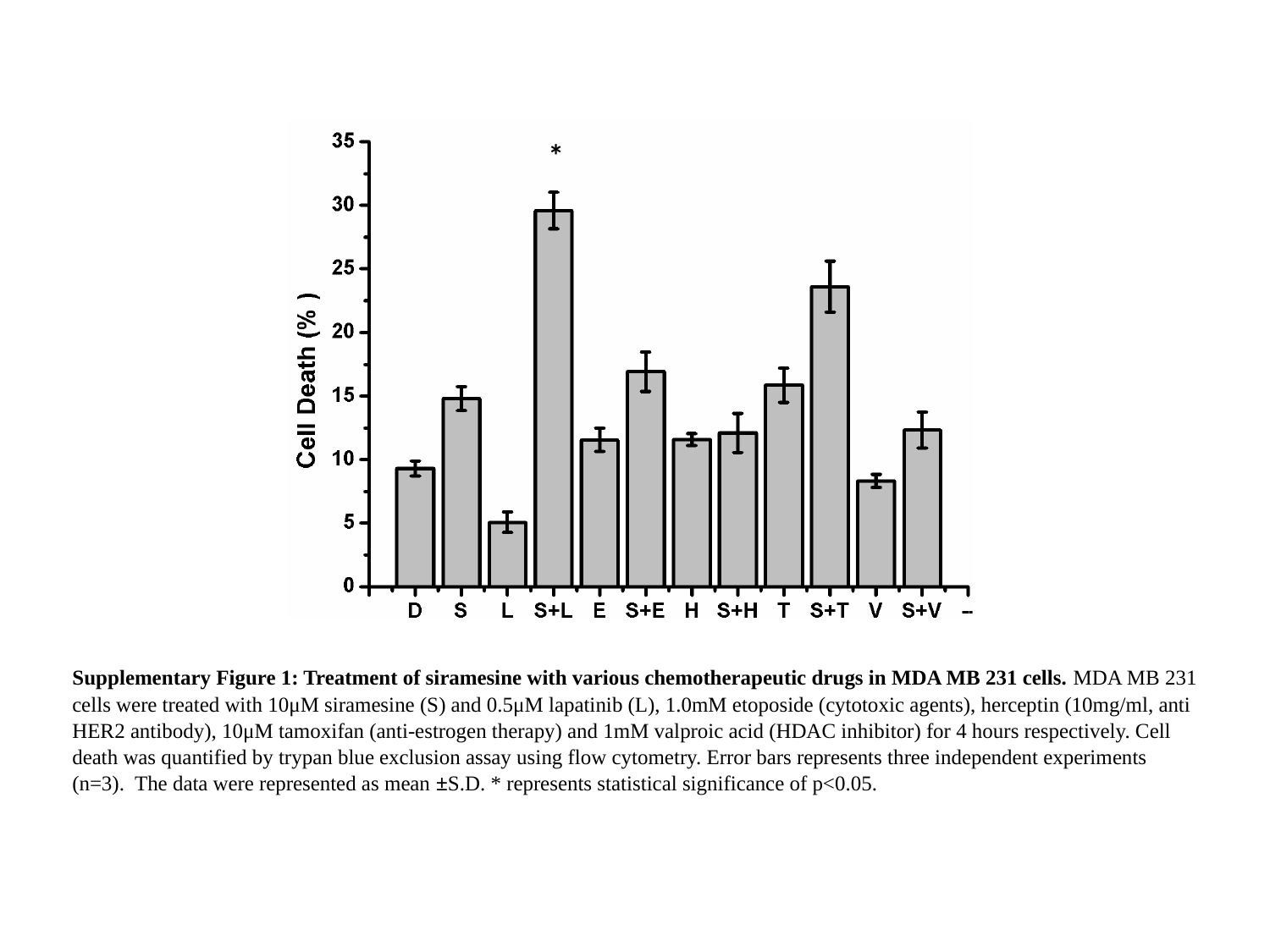

*
Supplementary Figure 1: Treatment of siramesine with various chemotherapeutic drugs in MDA MB 231 cells. MDA MB 231 cells were treated with 10μM siramesine (S) and 0.5μM lapatinib (L), 1.0mM etoposide (cytotoxic agents), herceptin (10mg/ml, anti HER2 antibody), 10μM tamoxifan (anti-estrogen therapy) and 1mM valproic acid (HDAC inhibitor) for 4 hours respectively. Cell death was quantified by trypan blue exclusion assay using flow cytometry. Error bars represents three independent experiments (n=3). The data were represented as mean ±S.D. * represents statistical significance of p<0.05.

## Slide 2
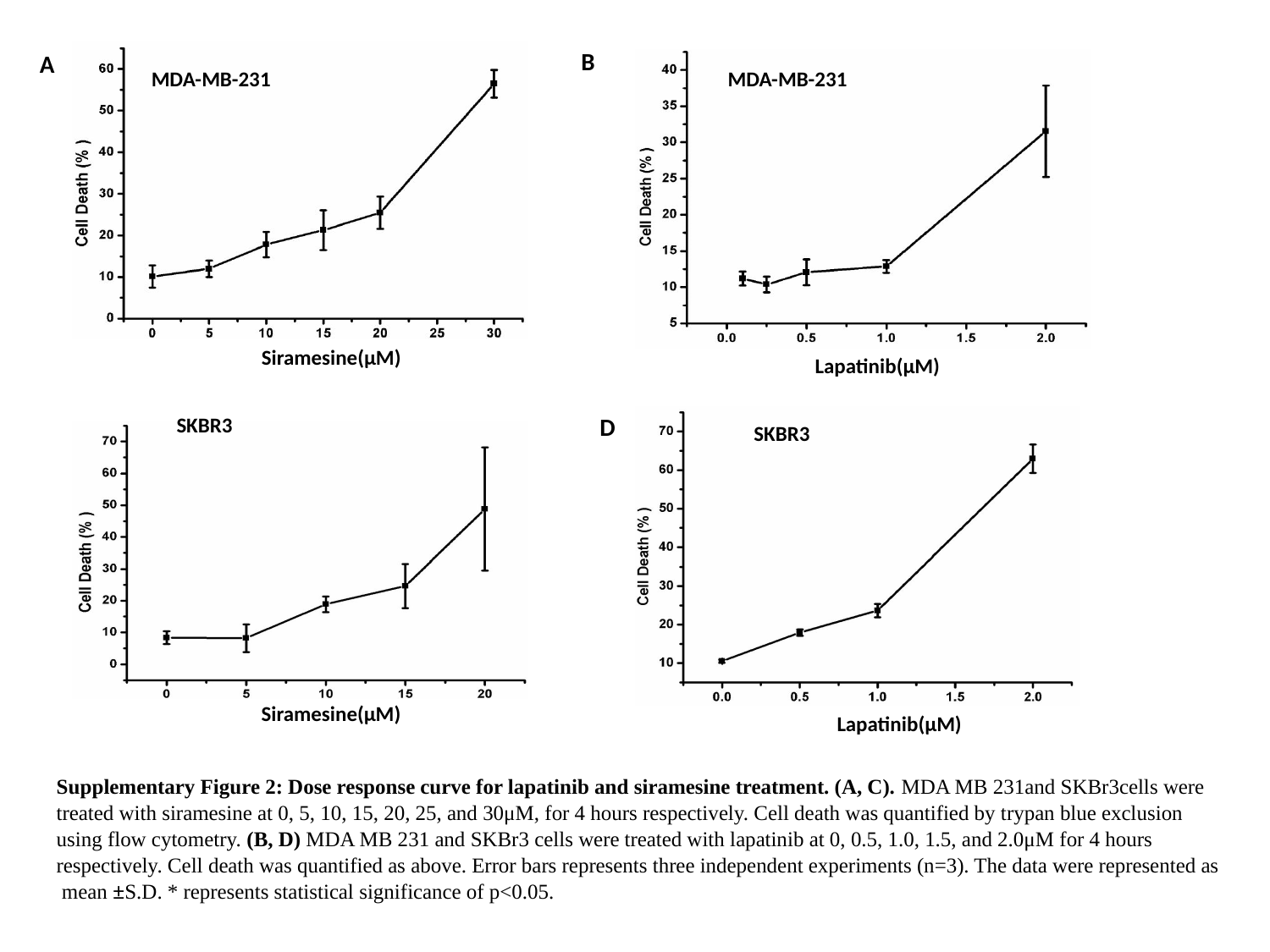

B
A
MDA-MB-231
 Lapatinib(µM)
MDA-MB-231
SKBR3
Siramesine(µM)
D
SKBR3
Lapatinib(µM)
Siramesine(µM)
Supplementary Figure 2: Dose response curve for lapatinib and siramesine treatment. (A, C). MDA MB 231and SKBr3cells were treated with siramesine at 0, 5, 10, 15, 20, 25, and 30μM, for 4 hours respectively. Cell death was quantified by trypan blue exclusion using flow cytometry. (B, D) MDA MB 231 and SKBr3 cells were treated with lapatinib at 0, 0.5, 1.0, 1.5, and 2.0μM for 4 hours respectively. Cell death was quantified as above. Error bars represents three independent experiments (n=3). The data were represented as mean ±S.D. * represents statistical significance of p<0.05.

## Slide 3
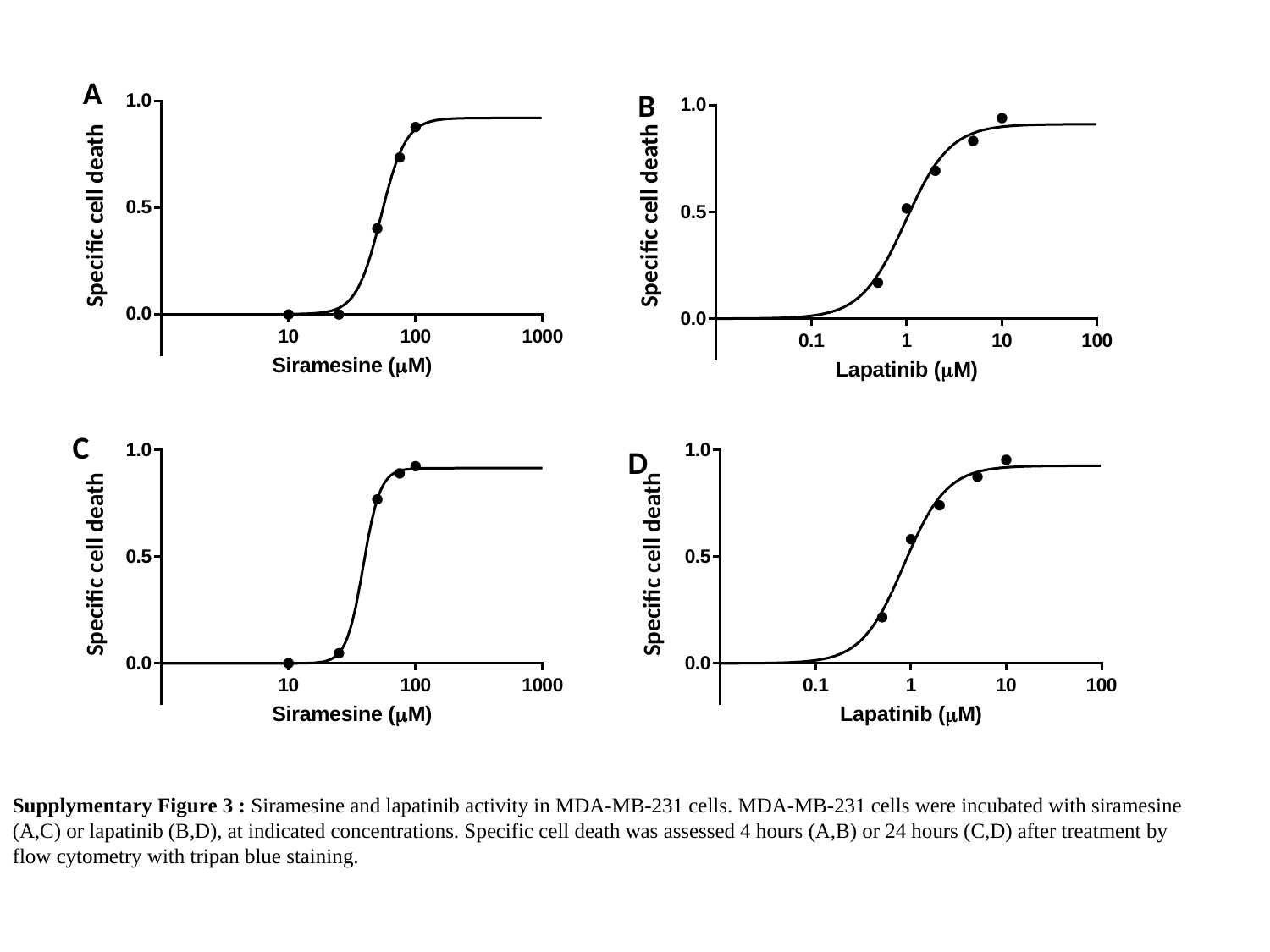

A
B
Specific cell death
Specific cell death
C
D
Specific cell death
Specific cell death
Supplymentary Figure 3 : Siramesine and lapatinib activity in MDA-MB-231 cells. MDA-MB-231 cells were incubated with siramesine (A,C) or lapatinib (B,D), at indicated concentrations. Specific cell death was assessed 4 hours (A,B) or 24 hours (C,D) after treatment by flow cytometry with tripan blue staining.

## Slide 4
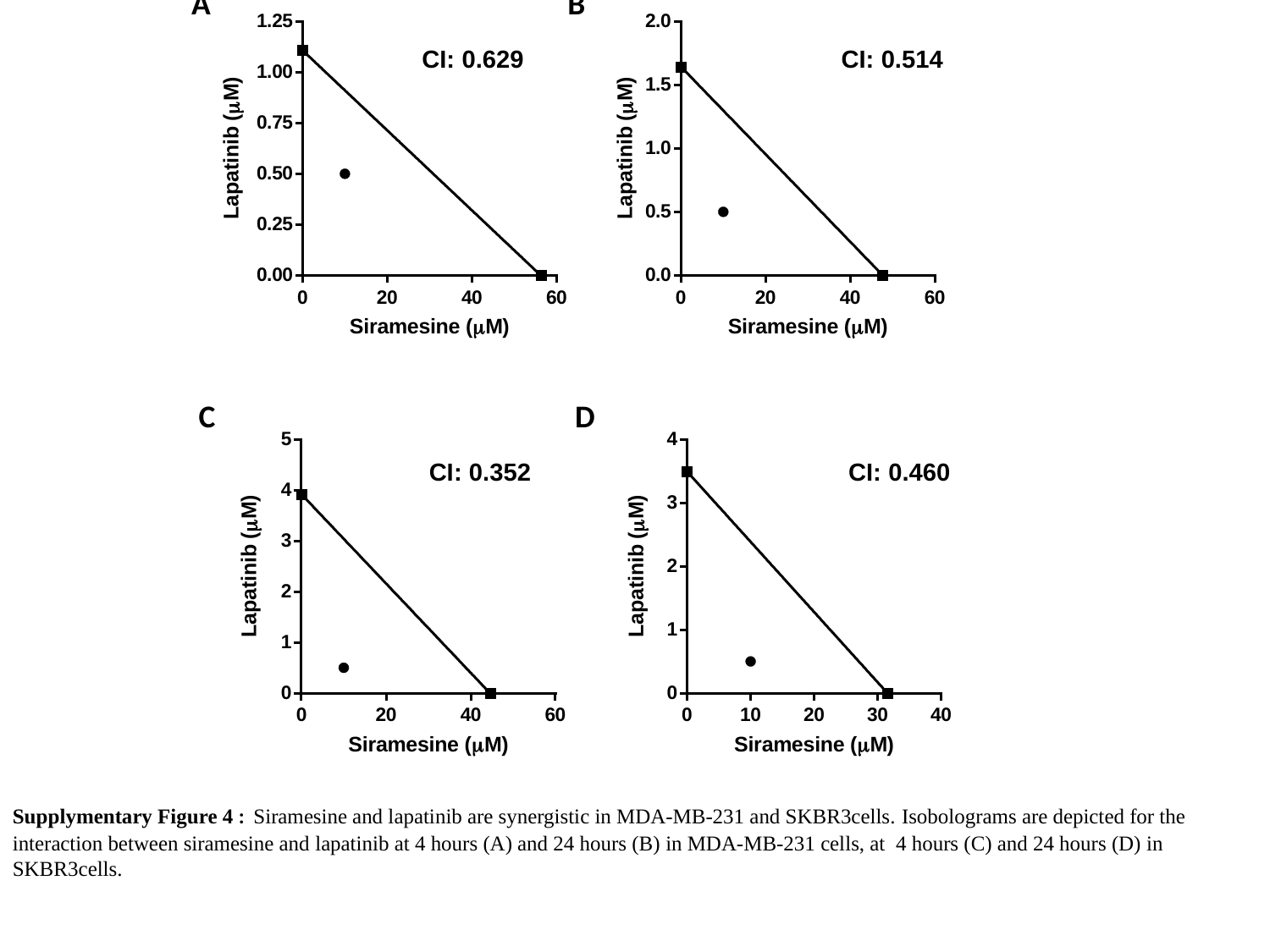

A
B
CI: 0.629
CI: 0.514
C
D
CI: 0.352
CI: 0.460
Supplymentary Figure 4 : Siramesine and lapatinib are synergistic in MDA-MB-231 and SKBR3cells. Isobolograms are depicted for the interaction between siramesine and lapatinib at 4 hours (A) and 24 hours (B) in MDA-MB-231 cells, at 4 hours (C) and 24 hours (D) in SKBR3cells.

## Slide 5
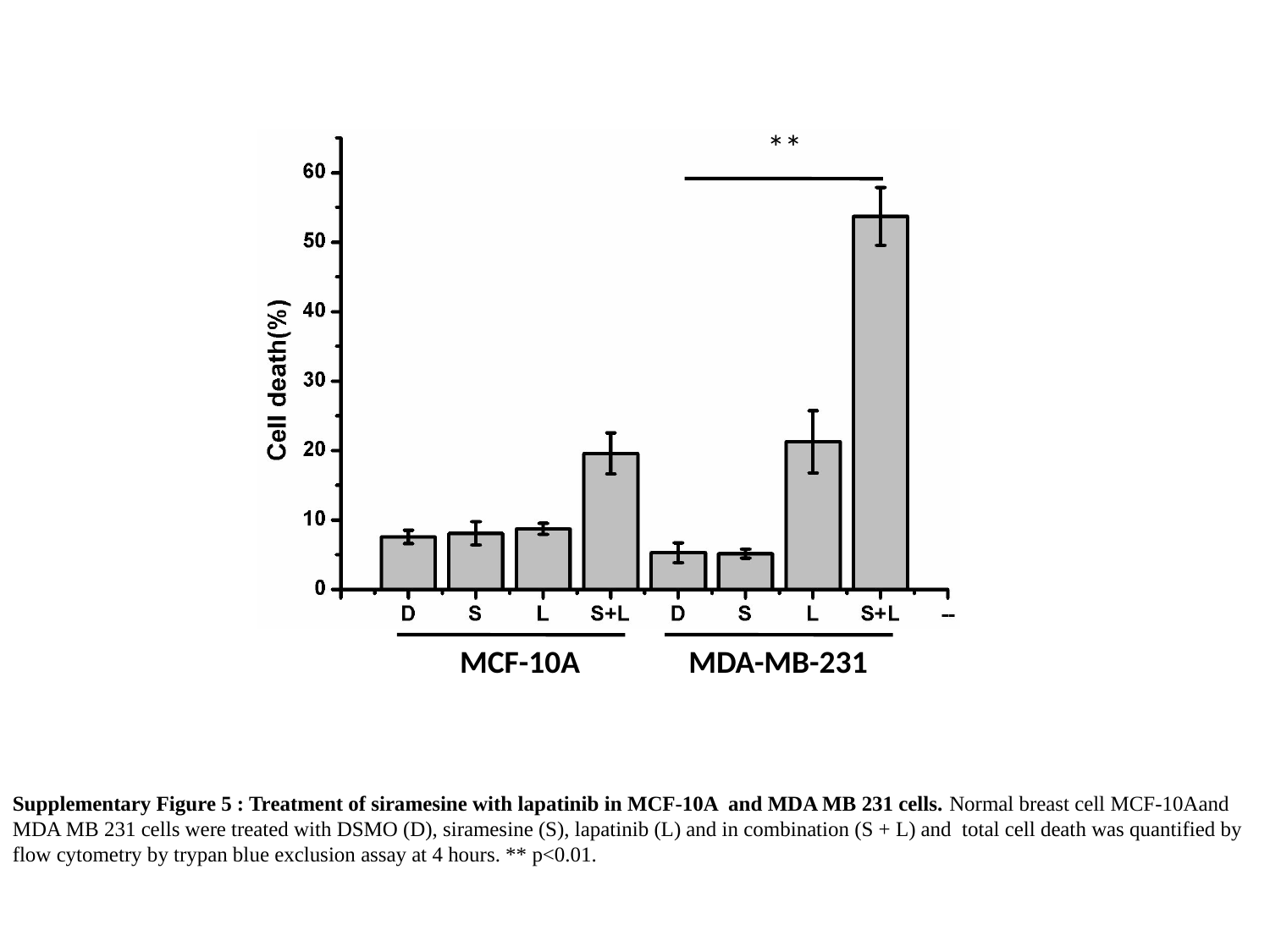

**
MCF-10A
MDA-MB-231
Supplementary Figure 5 : Treatment of siramesine with lapatinib in MCF-10A and MDA MB 231 cells. Normal breast cell MCF-10Aand MDA MB 231 cells were treated with DSMO (D), siramesine (S), lapatinib (L) and in combination (S + L) and total cell death was quantified by flow cytometry by trypan blue exclusion assay at 4 hours. ** p<0.01.

## Slide 6
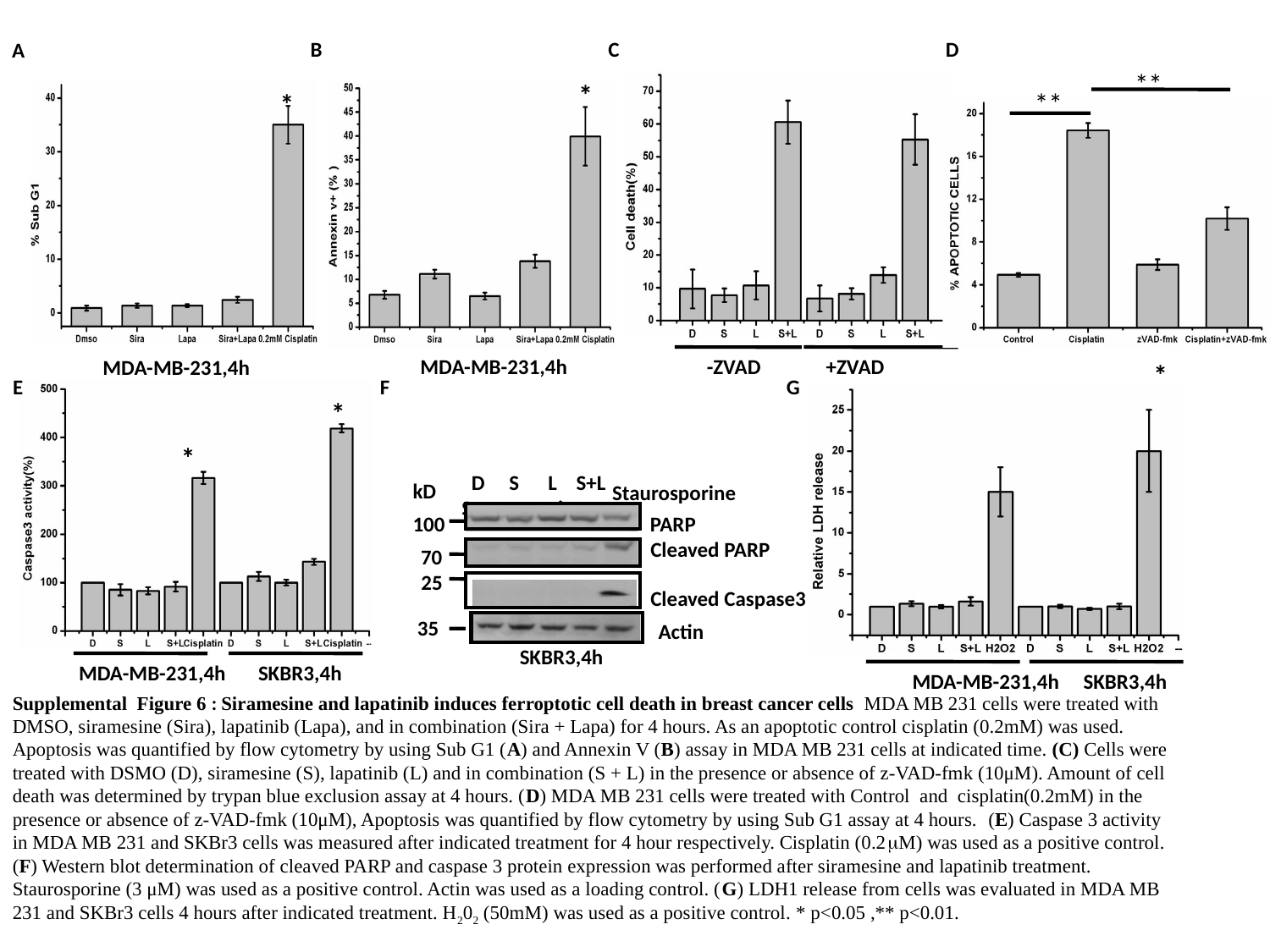

B
C
A
*
*
MDA-MB-231,4h
-ZVAD
+ZVAD
MDA-MB-231,4h
 *
E
F
G
*
*
 *
 D S L S+L Staurosporine
kD
100
PARP
Cleaved PARP
70
25
Cleaved Caspase3
35
Actin
SKBR3,4h
MDA-MB-231,4h
SKBR3,4h
MDA-MB-231,4h
SKBR3,4h
D
**
**
Staurosporine
Supplemental Figure 6 : Siramesine and lapatinib induces ferroptotic cell death in breast cancer cells MDA MB 231 cells were treated with DMSO, siramesine (Sira), lapatinib (Lapa), and in combination (Sira + Lapa) for 4 hours. As an apoptotic control cisplatin (0.2mM) was used. Apoptosis was quantified by flow cytometry by using Sub G1 (A) and Annexin V (B) assay in MDA MB 231 cells at indicated time. (C) Cells were treated with DSMO (D), siramesine (S), lapatinib (L) and in combination (S + L) in the presence or absence of z-VAD-fmk (10μM). Amount of cell death was determined by trypan blue exclusion assay at 4 hours. (D) MDA MB 231 cells were treated with Control and cisplatin(0.2mM) in the presence or absence of z-VAD-fmk (10μM), Apoptosis was quantified by flow cytometry by using Sub G1 assay at 4 hours. (E) Caspase 3 activity in MDA MB 231 and SKBr3 cells was measured after indicated treatment for 4 hour respectively. Cisplatin (0.2μM) was used as a positive control. (F) Western blot determination of cleaved PARP and caspase 3 protein expression was performed after siramesine and lapatinib treatment. Staurosporine (3 μM) was used as a positive control. Actin was used as a loading control. (G) LDH1 release from cells was evaluated in MDA MB 231 and SKBr3 cells 4 hours after indicated treatment. H202 (50mM) was used as a positive control. * p<0.05 ,** p<0.01.

## Slide 7
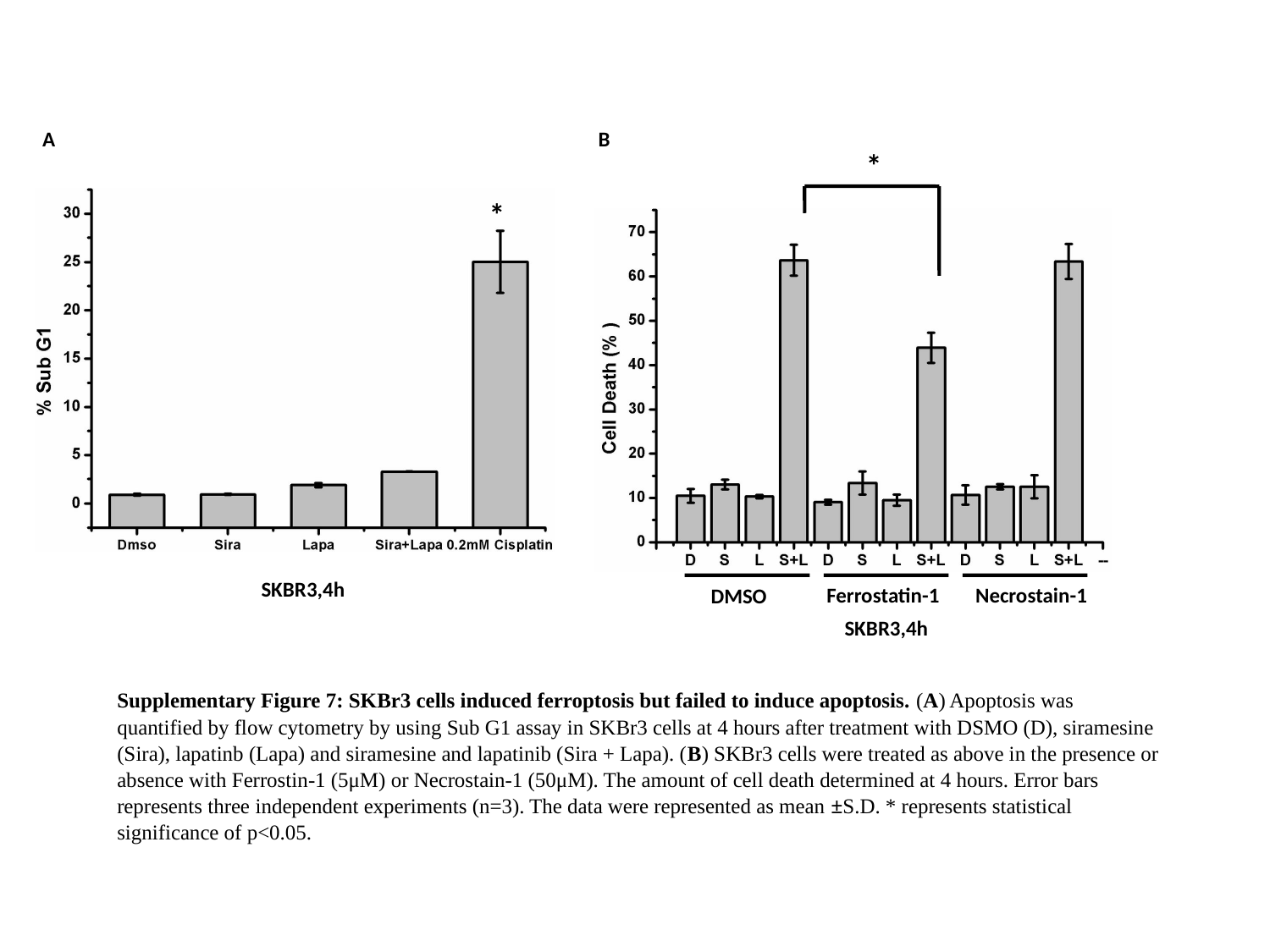

A
B
*
*
SKBR3,4h
Ferrostatin-1
Necrostain-1
DMSO
SKBR3,4h
Supplementary Figure 7: SKBr3 cells induced ferroptosis but failed to induce apoptosis. (A) Apoptosis was quantified by flow cytometry by using Sub G1 assay in SKBr3 cells at 4 hours after treatment with DSMO (D), siramesine (Sira), lapatinb (Lapa) and siramesine and lapatinib (Sira + Lapa). (B) SKBr3 cells were treated as above in the presence or absence with Ferrostin-1 (5μM) or Necrostain-1 (50μM). The amount of cell death determined at 4 hours. Error bars represents three independent experiments (n=3). The data were represented as mean ±S.D. * represents statistical significance of p<0.05.

## Slide 8
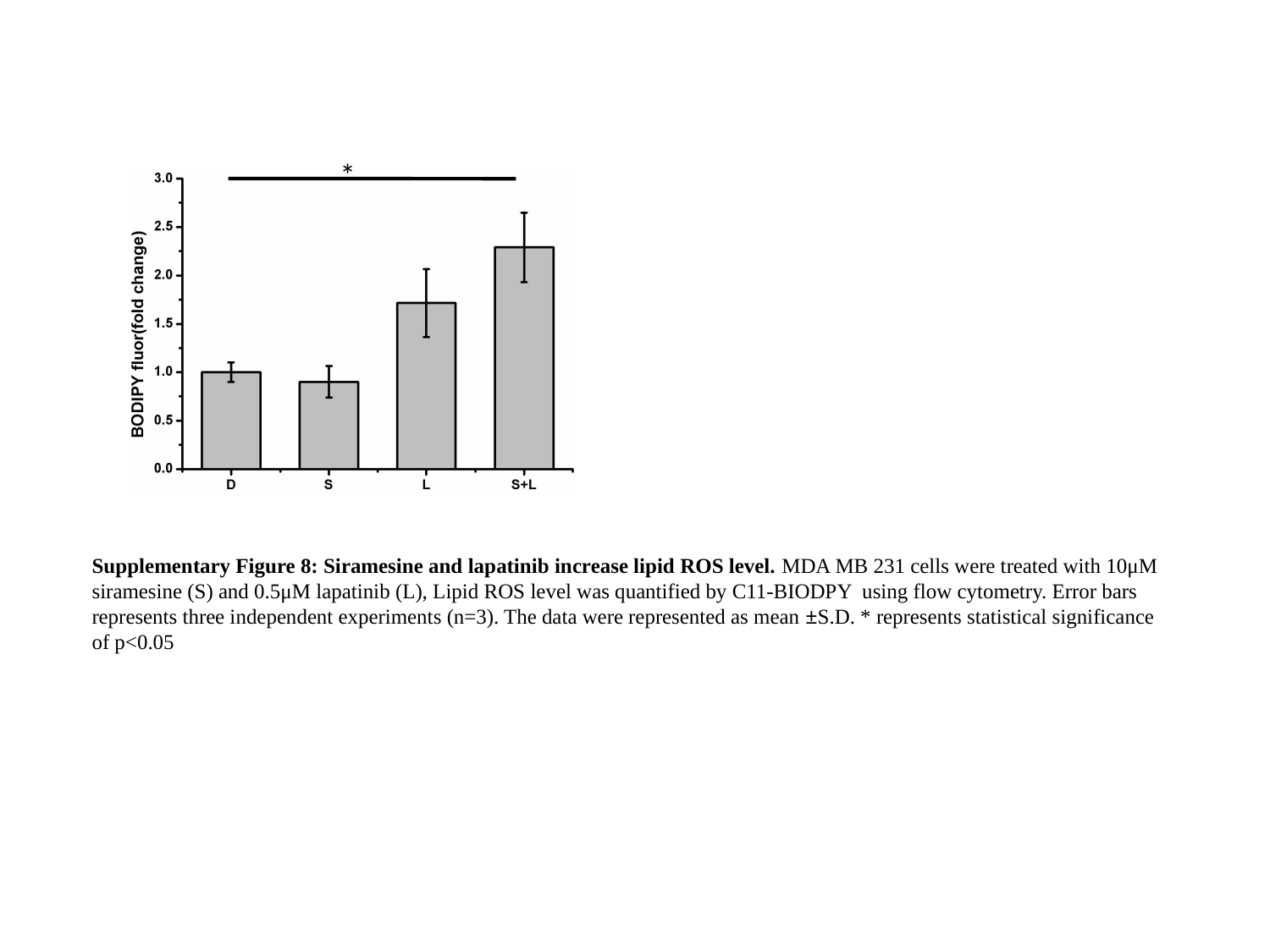

*
Supplementary Figure 8: Siramesine and lapatinib increase lipid ROS level. MDA MB 231 cells were treated with 10μM siramesine (S) and 0.5μM lapatinib (L), Lipid ROS level was quantified by C11-BIODPY using flow cytometry. Error bars represents three independent experiments (n=3). The data were represented as mean ±S.D. * represents statistical significance of p<0.05

## Slide 9
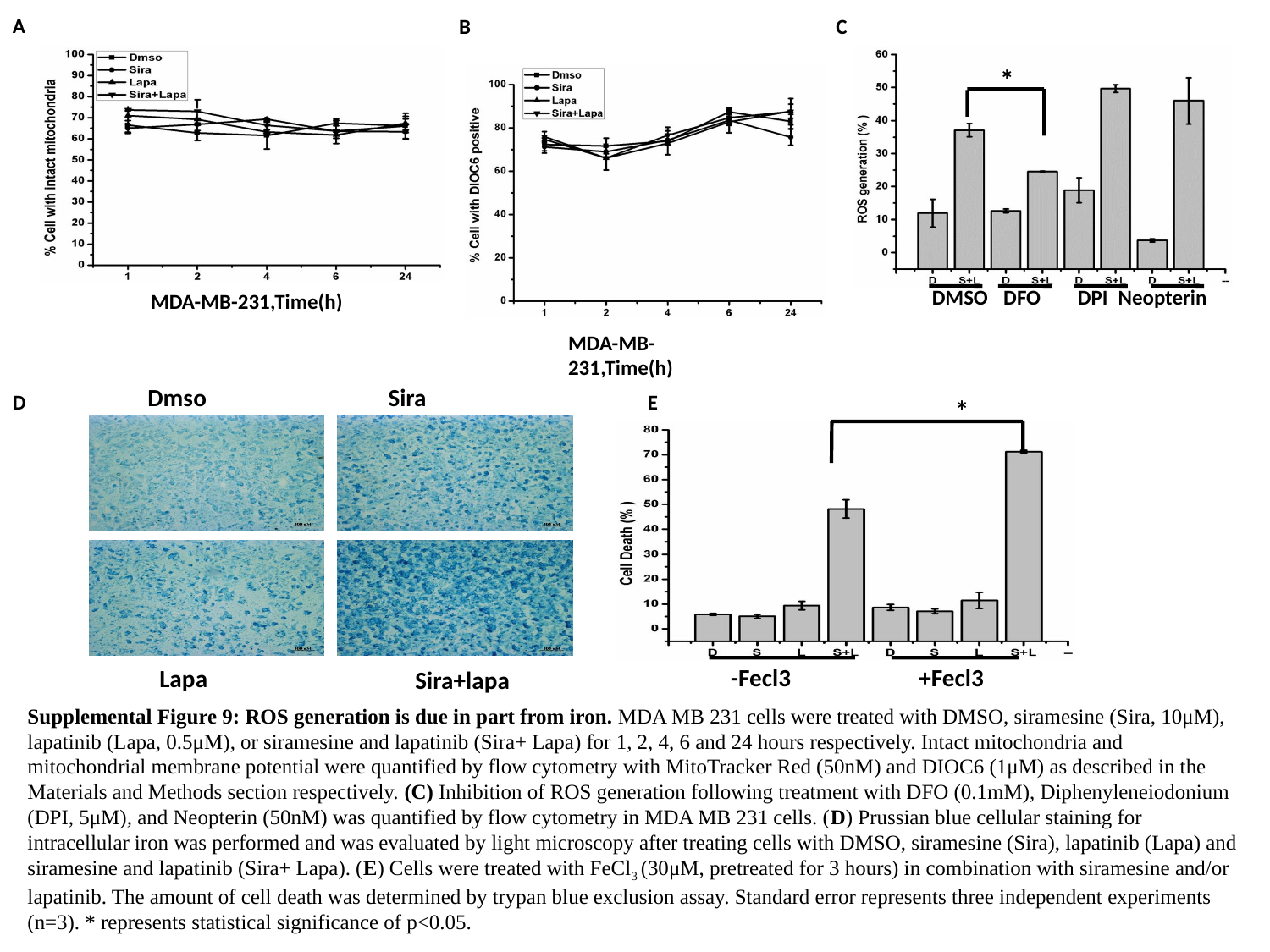

A
B
C
MDA-MB-231,Time(h)
*
DMSO
DFO
DPI
Neopterin
MDA-MB-231,Time(h)
 Sira
Dmso
D
E
*
-Fecl3
+Fecl3
Lapa
 Sira+lapa
Supplemental Figure 9: ROS generation is due in part from iron. MDA MB 231 cells were treated with DMSO, siramesine (Sira, 10μM), lapatinib (Lapa, 0.5μM), or siramesine and lapatinib (Sira+ Lapa) for 1, 2, 4, 6 and 24 hours respectively. Intact mitochondria and mitochondrial membrane potential were quantified by flow cytometry with MitoTracker Red (50nM) and DIOC6 (1μM) as described in the Materials and Methods section respectively. (C) Inhibition of ROS generation following treatment with DFO (0.1mM), Diphenyleneiodonium (DPI, 5μM), and Neopterin (50nM) was quantified by flow cytometry in MDA MB 231 cells. (D) Prussian blue cellular staining for intracellular iron was performed and was evaluated by light microscopy after treating cells with DMSO, siramesine (Sira), lapatinib (Lapa) and siramesine and lapatinib (Sira+ Lapa). (E) Cells were treated with FeCl3 (30μM, pretreated for 3 hours) in combination with siramesine and/or lapatinib. The amount of cell death was determined by trypan blue exclusion assay. Standard error represents three independent experiments (n=3). * represents statistical significance of p<0.05.

## Slide 10
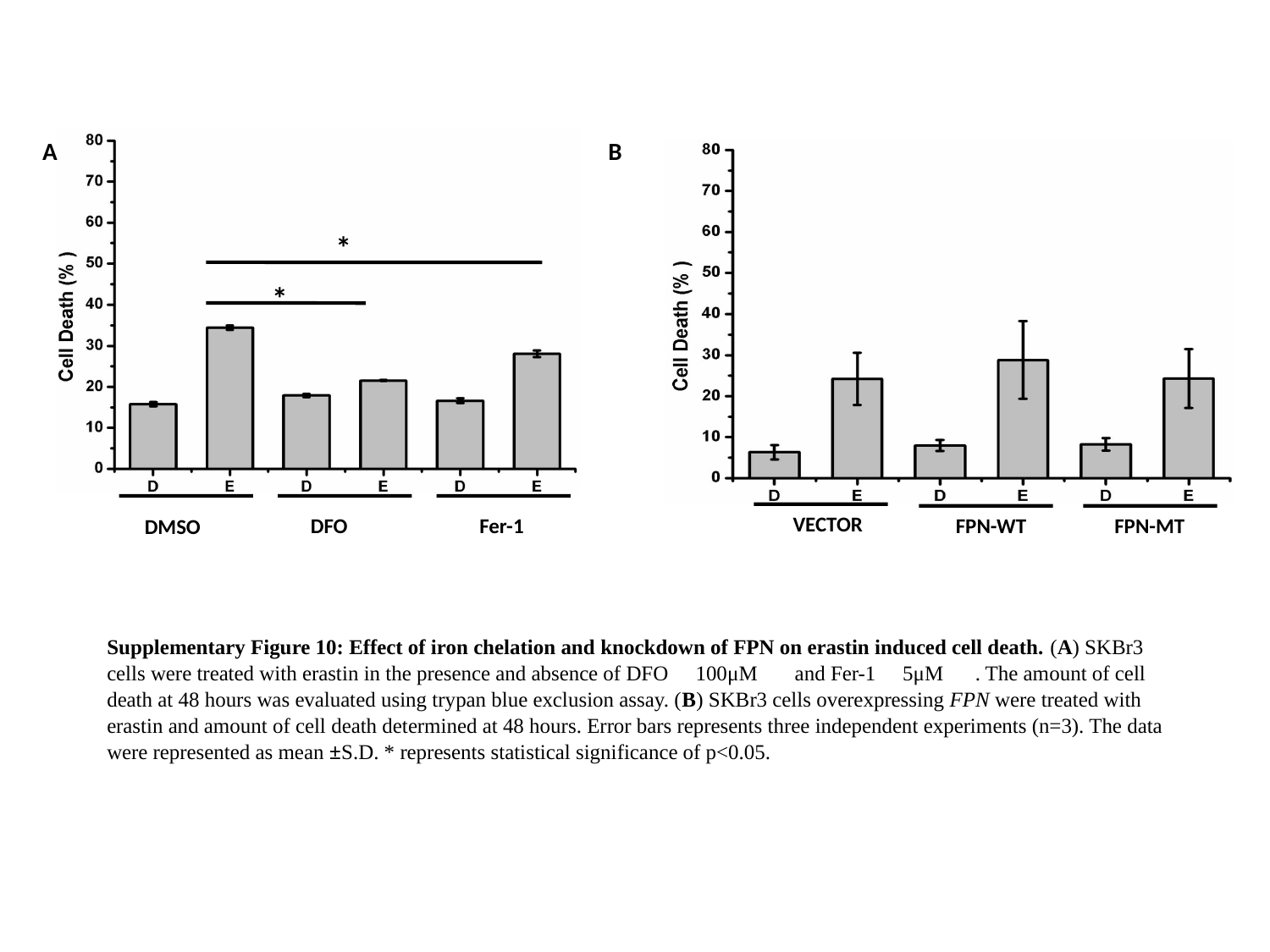

A
*
*
B
VECTOR
DFO
Fer-1
FPN-WT
FPN-MT
DMSO
Supplementary Figure 10: Effect of iron chelation and knockdown of FPN on erastin induced cell death. (A) SKBr3 cells were treated with erastin in the presence and absence of DFO（100μM ） and Fer-1（5μM ）. The amount of cell death at 48 hours was evaluated using trypan blue exclusion assay. (B) SKBr3 cells overexpressing FPN were treated with erastin and amount of cell death determined at 48 hours. Error bars represents three independent experiments (n=3). The data were represented as mean ±S.D. * represents statistical significance of p<0.05.

## Slide 11
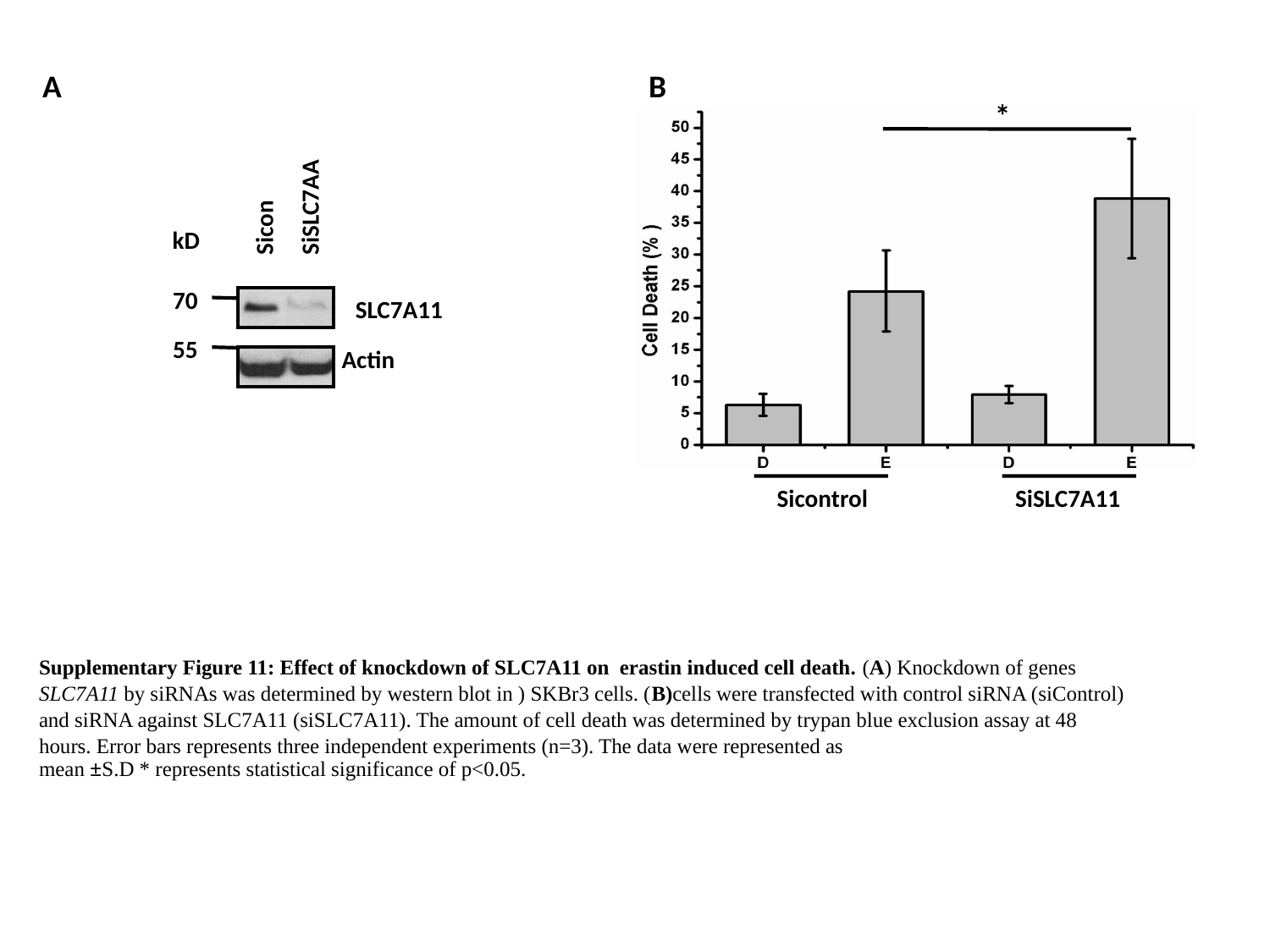

*
Sicontrol
SiSLC7A11
A
B
Sicon
SiSLC7AA
kD
70
SLC7A11
55
Actin
Supplementary Figure 11: Effect of knockdown of SLC7A11 on erastin induced cell death. (A) Knockdown of genes SLC7A11 by siRNAs was determined by western blot in ) SKBr3 cells. (B)cells were transfected with control siRNA (siControl) and siRNA against SLC7A11 (siSLC7A11). The amount of cell death was determined by trypan blue exclusion assay at 48 hours. Error bars represents three independent experiments (n=3). The data were represented as
mean ±S.D * represents statistical significance of p<0.05.

## Slide 12
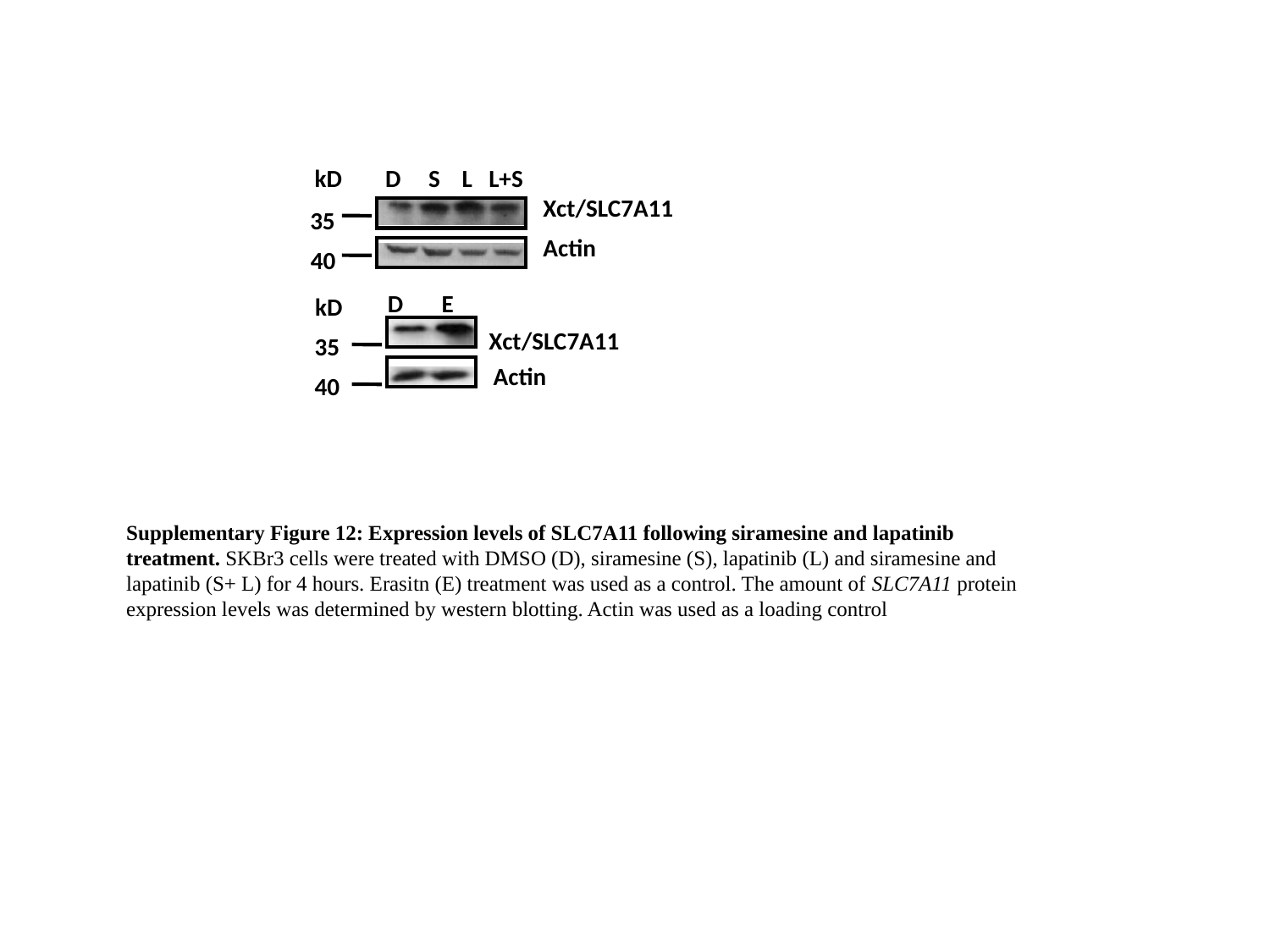

kD
 D S L L+S
Xct/SLC7A11
35
Actin
40
D
E
kD
Xct/SLC7A11
35
Actin
40
Supplementary Figure 12: Expression levels of SLC7A11 following siramesine and lapatinib treatment. SKBr3 cells were treated with DMSO (D), siramesine (S), lapatinib (L) and siramesine and lapatinib (S+ L) for 4 hours. Erasitn (E) treatment was used as a control. The amount of SLC7A11 protein expression levels was determined by western blotting. Actin was used as a loading control

## Slide 13
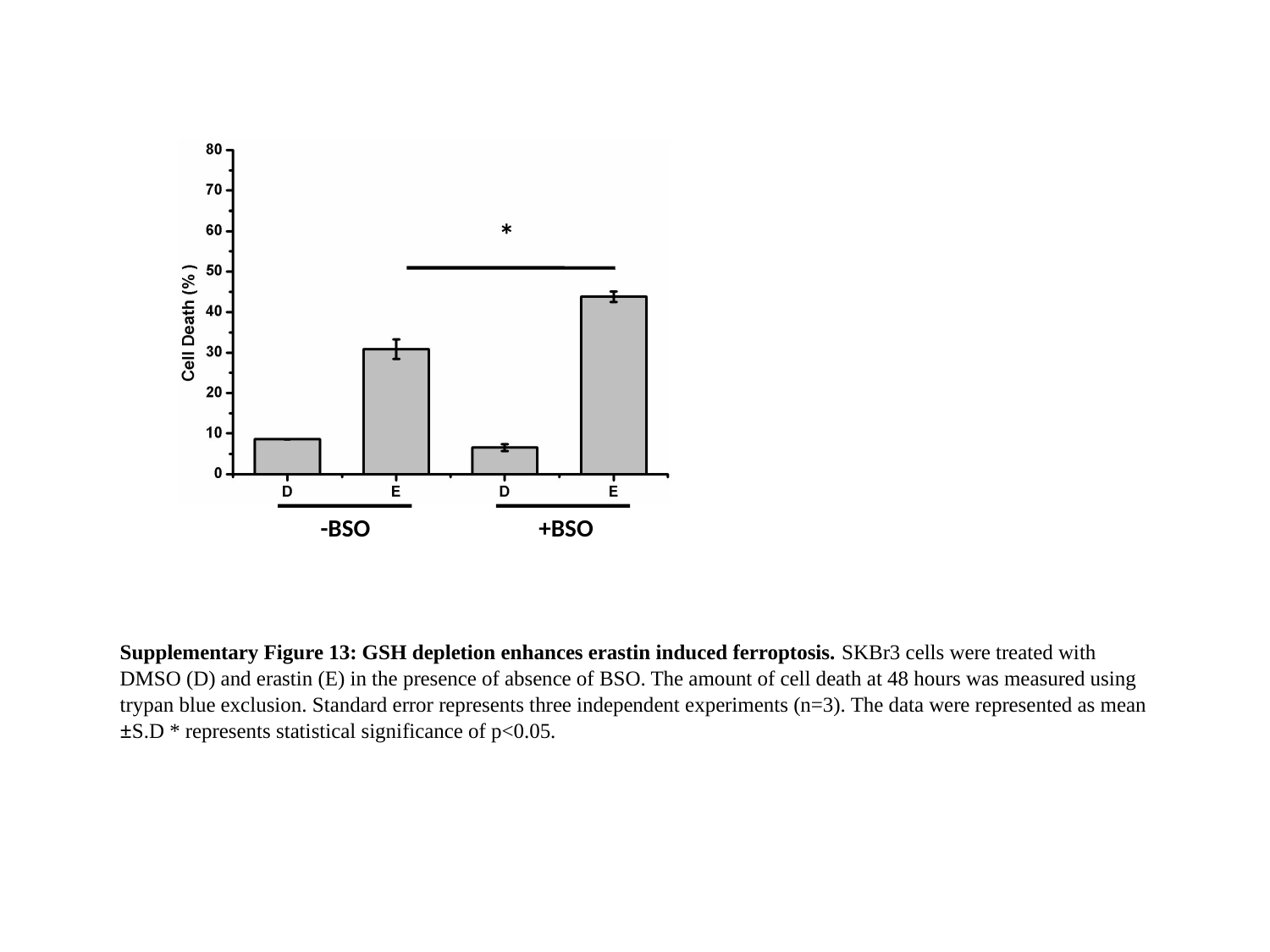

*
-BSO
+BSO
Supplementary Figure 13: GSH depletion enhances erastin induced ferroptosis. SKBr3 cells were treated with DMSO (D) and erastin (E) in the presence of absence of BSO. The amount of cell death at 48 hours was measured using trypan blue exclusion. Standard error represents three independent experiments (n=3). The data were represented as mean ±S.D * represents statistical significance of p<0.05.

## Slide 14
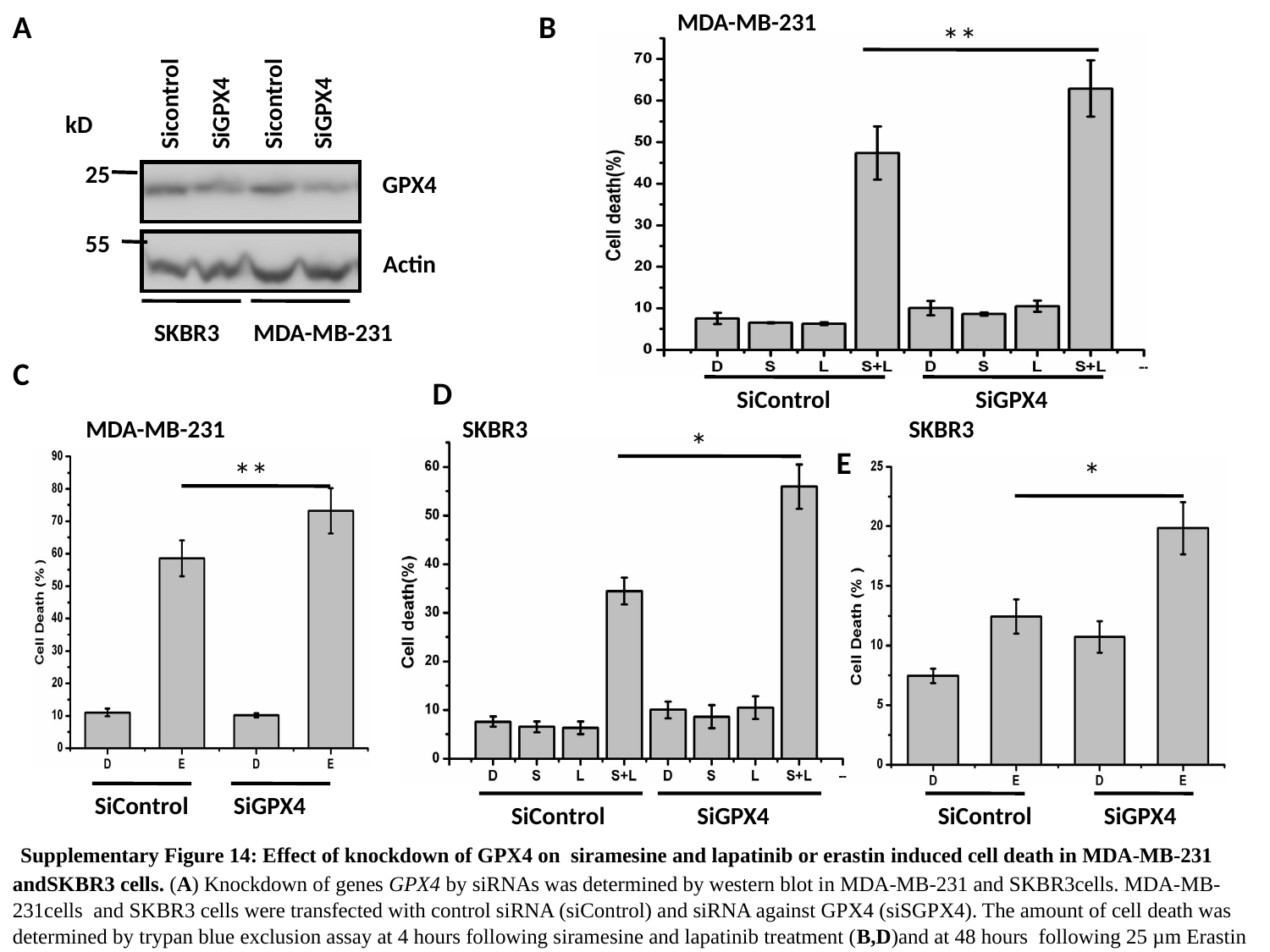

A
B
MDA-MB-231
**
SiControl
SiGPX4
SiGPX4
SiGPX4
Sicontrol
Sicontrol
kD
25
GPX4
Actin
SKBR3
MDA-MB-231
55
C
D
MDA-MB-231
*
*
SiControl
SiGPX4
SiControl
SiGPX4
E
**
SiControl
SiGPX4
SKBR3
SKBR3
 Supplementary Figure 14: Effect of knockdown of GPX4 on siramesine and lapatinib or erastin induced cell death in MDA-MB-231 andSKBR3 cells. (A) Knockdown of genes GPX4 by siRNAs was determined by western blot in MDA-MB-231 and SKBR3cells. MDA-MB-231cells and SKBR3 cells were transfected with control siRNA (siControl) and siRNA against GPX4 (siSGPX4). The amount of cell death was determined by trypan blue exclusion assay at 4 hours following siramesine and lapatinib treatment (B,D)and at 48 hours following 25 µm Erastin treatment(C,E). Error bars represents three independent experiments (n=3). The data were represented as mean ±S.D * represents statistical significance of p<0.05.

## Slide 15
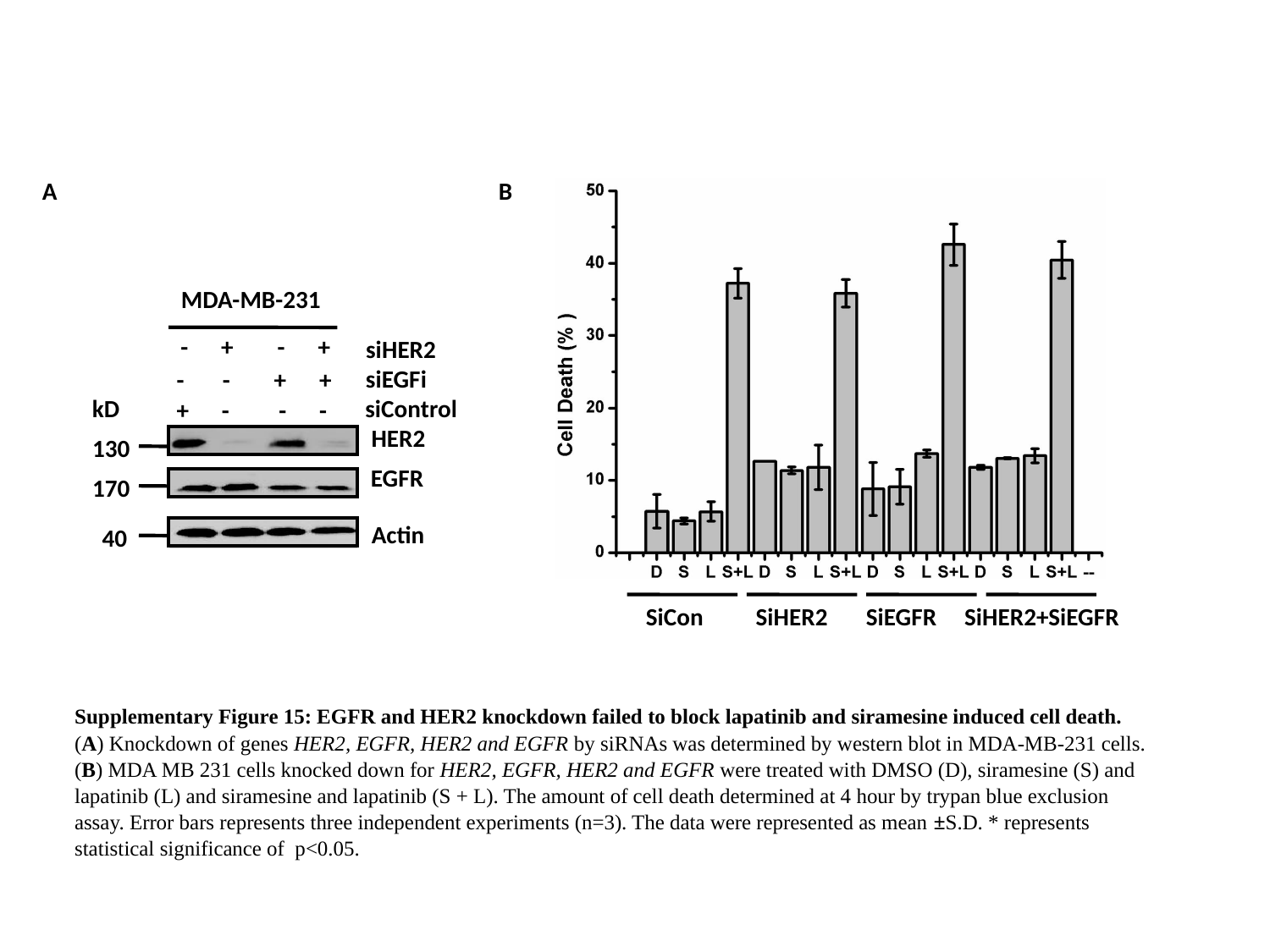

A
B
MDA-MB-231
 - + - +
 - - + +
 + - - -
 siHER2
 siEGFi
kD
130
170
40
 siControl
 HER2
 EGFR
 Actin
SiCon
SiHER2
SiEGFR
SiHER2+SiEGFR
Supplementary Figure 15: EGFR and HER2 knockdown failed to block lapatinib and siramesine induced cell death. (A) Knockdown of genes HER2, EGFR, HER2 and EGFR by siRNAs was determined by western blot in MDA-MB-231 cells. (B) MDA MB 231 cells knocked down for HER2, EGFR, HER2 and EGFR were treated with DMSO (D), siramesine (S) and lapatinib (L) and siramesine and lapatinib (S + L). The amount of cell death determined at 4 hour by trypan blue exclusion assay. Error bars represents three independent experiments (n=3). The data were represented as mean ±S.D. * represents statistical significance of p<0.05.

## Slide 16
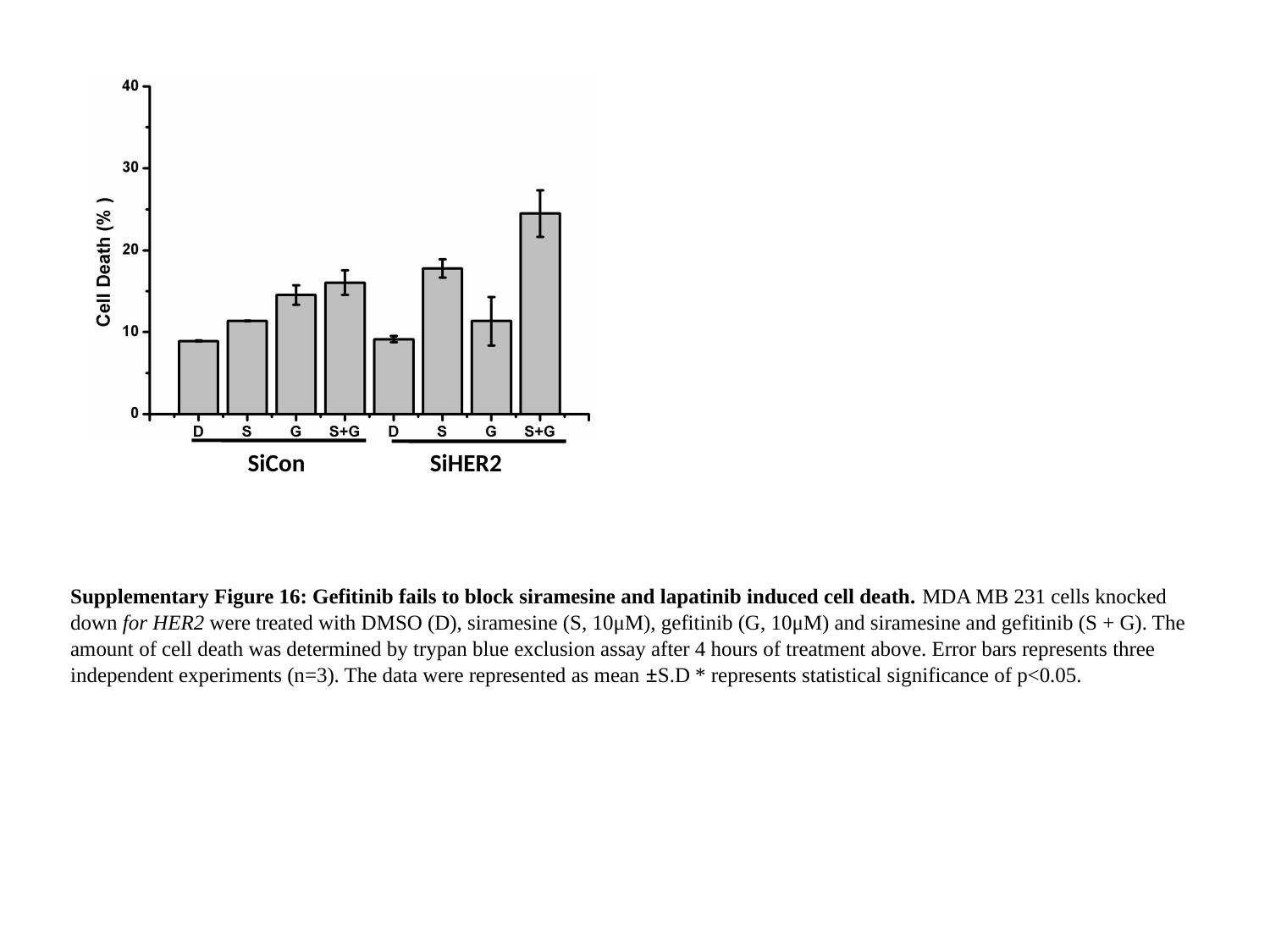

SiCon
SiHER2
Supplementary Figure 16: Gefitinib fails to block siramesine and lapatinib induced cell death. MDA MB 231 cells knocked down for HER2 were treated with DMSO (D), siramesine (S, 10μM), gefitinib (G, 10μM) and siramesine and gefitinib (S + G). The amount of cell death was determined by trypan blue exclusion assay after 4 hours of treatment above. Error bars represents three independent experiments (n=3). The data were represented as mean ±S.D * represents statistical significance of p<0.05.

## Slide 17
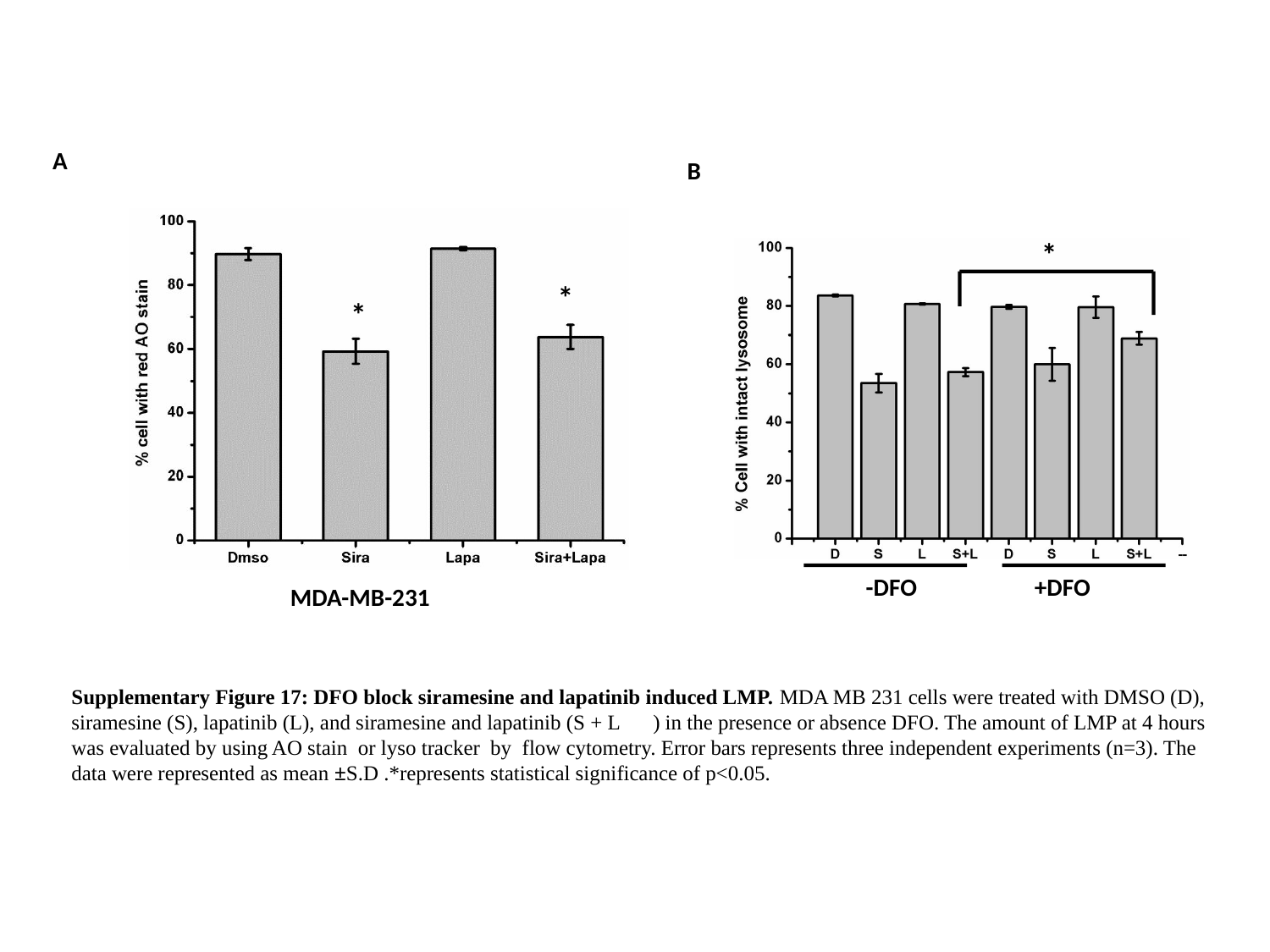

A
B
*
-DFO
+DFO
*
*
MDA-MB-231
Supplementary Figure 17: DFO block siramesine and lapatinib induced LMP. MDA MB 231 cells were treated with DMSO (D), siramesine (S), lapatinib (L), and siramesine and lapatinib (S + L） ) in the presence or absence DFO. The amount of LMP at 4 hours was evaluated by using AO stain or lyso tracker by flow cytometry. Error bars represents three independent experiments (n=3). The data were represented as mean ±S.D .*represents statistical significance of p<0.05.

## Slide 18
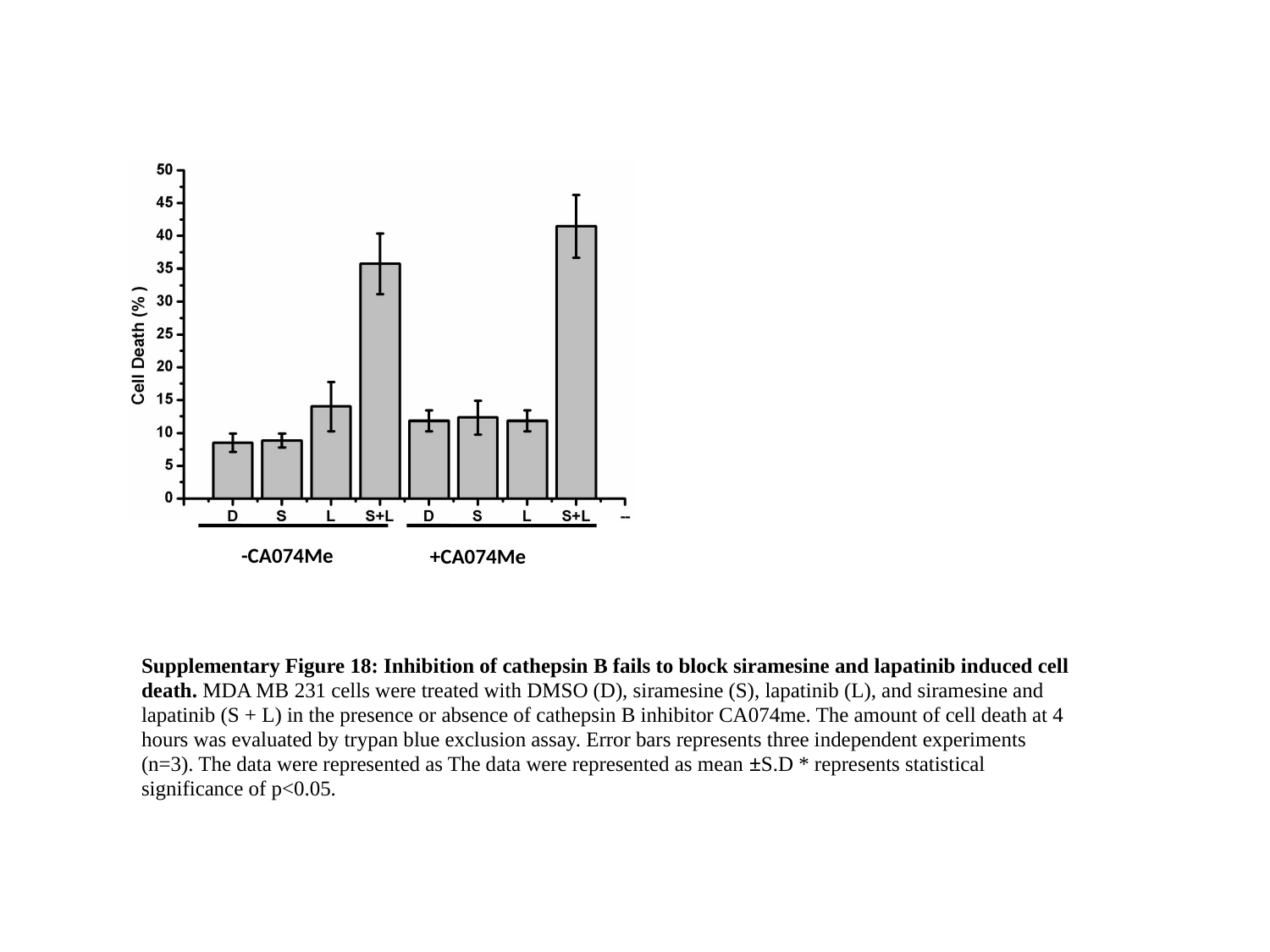

-CA074Me
+CA074Me
Supplementary Figure 18: Inhibition of cathepsin B fails to block siramesine and lapatinib induced cell death. MDA MB 231 cells were treated with DMSO (D), siramesine (S), lapatinib (L), and siramesine and lapatinib (S + L) in the presence or absence of cathepsin B inhibitor CA074me. The amount of cell death at 4 hours was evaluated by trypan blue exclusion assay. Error bars represents three independent experiments (n=3). The data were represented as The data were represented as mean ±S.D * represents statistical significance of p<0.05.

## Slide 19
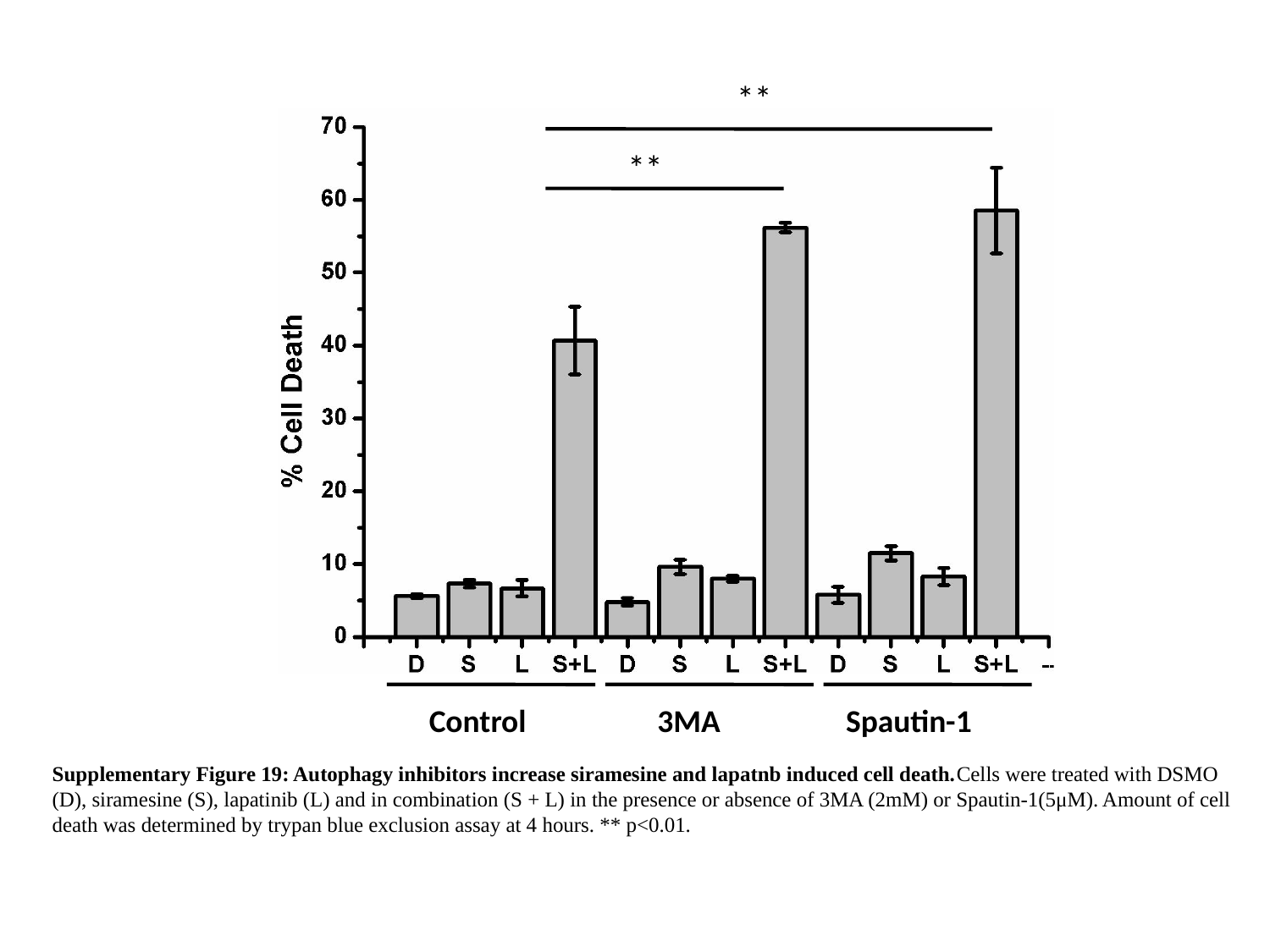

**
**
Control
3MA
Spautin-1
Supplementary Figure 19: Autophagy inhibitors increase siramesine and lapatnb induced cell death.Cells were treated with DSMO (D), siramesine (S), lapatinib (L) and in combination (S + L) in the presence or absence of 3MA (2mM) or Spautin-1(5μM). Amount of cell death was determined by trypan blue exclusion assay at 4 hours. ** p<0.01.

## Slide 20
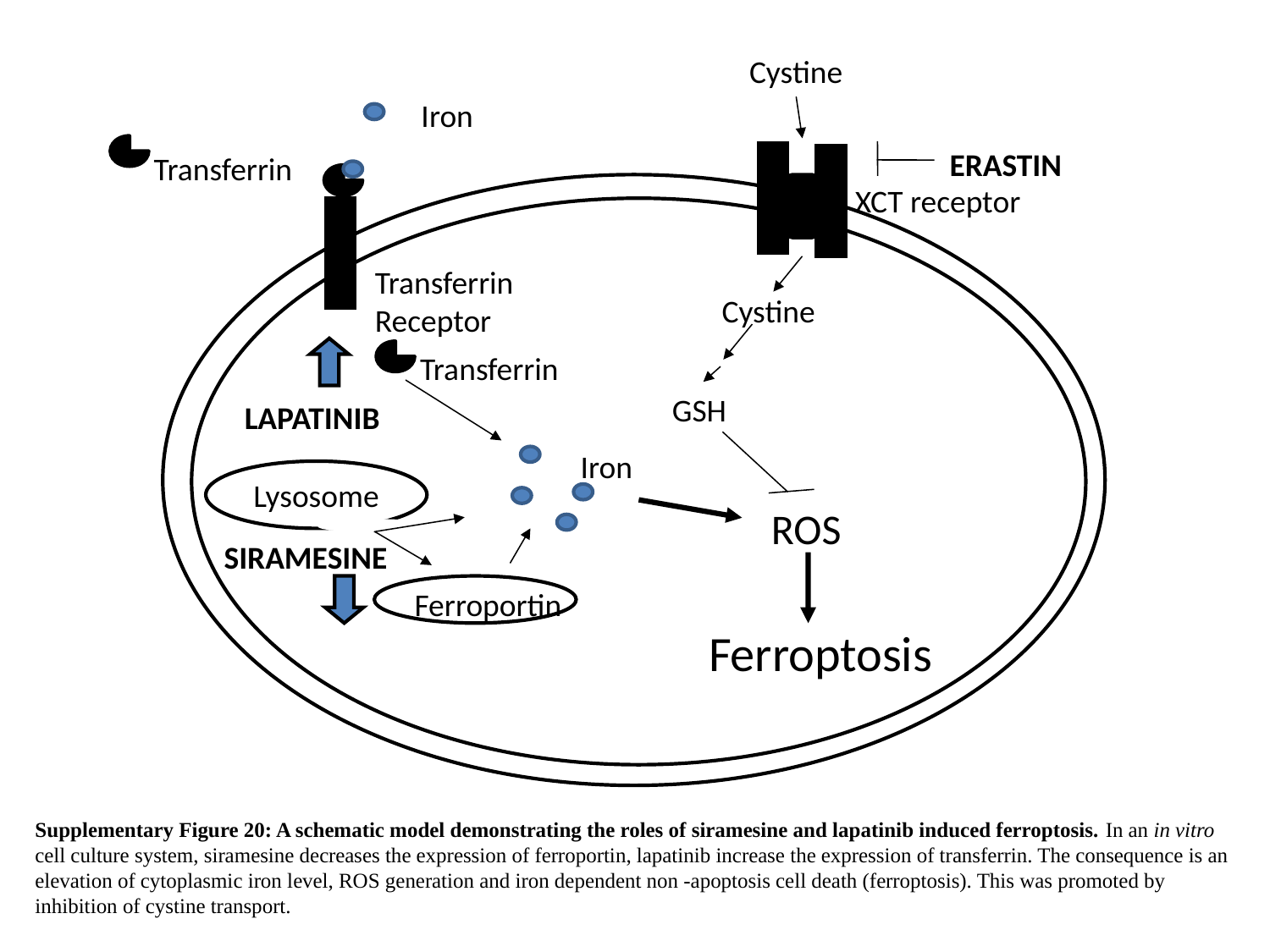

Cystine
Iron
ERASTIN
Transferrin
XCT receptor
Transferrin
Receptor
Cystine
Transferrin
GSH
LAPATINIB
Iron
Lysosome
ROS
SIRAMESINE
Ferroportin
Ferroptosis
Supplementary Figure 20: A schematic model demonstrating the roles of siramesine and lapatinib induced ferroptosis. In an in vitro cell culture system, siramesine decreases the expression of ferroportin, lapatinib increase the expression of transferrin. The consequence is an elevation of cytoplasmic iron level, ROS generation and iron dependent non -apoptosis cell death (ferroptosis). This was promoted by inhibition of cystine transport.
